# Design and synthesis of 1,2,3-triazole thiadiazole hybrids with in vitro and in silico evaluation of their anti-inflammatory and anti-alzheimer activities

**DOI:** 10.1038/s41598-025-26140-8

**Published:** 2025-11-21

**Authors:** Ahmed R. Rabee, Hamida Abdel-Hamid, Saied M. Soliman, Samah Ashraf, Ahmed A. Sobhy, Doaa Ahmad Ghareeb, Aalaa K. Saad, Mohamed Hagar

**Affiliations:** 1 Chemistry Department, Faculty of Science, Alexandria University, P.O. Box 426, Alexandria, 21321 Egypt; 2 Bio-Screening and Preclinical Trial Lab, Biochemistry Department, Faculty of Science, Alexandria University, Alexandria, Egypt; 3 Microbiology and Immunology Department, Faculty of Pharmacy, Alexandria University, Alexandria, Egypt; 4 Center of Excellence for Drug Preclinical Studies (CE-DPS), Pharmaceutical and Fermentation Industry Development Center, City of Scientific Research & Technological Applications (SRTA-City), New Borg El Arab, Alexandria, Egypt; 5 Research Projects Unit, Pharos University in Alexandria, Canal El Mahmoudia Street, Beside Green Plaza Complex, Alexandria, 21648 Egypt

**Keywords:** 1,2,3-triazole, Thiadiazole, Chitosan nanoparticles, Molecular docking, DFT studies, Nanoscale materials, Computational chemistry, Nanoscience and technology, Nanoparticle synthesis

## Abstract

Nano technology possesses a role in the enhancement of anti-inflammatory and anti-Alzheimer activities of the synthesized triazole/thiadiazole hybrids **3a–c**. Selective propargylation of 5-amino-1,3,4-thiadiazole-2-thiol with propargyl bromide and triethyl amine followed by Click reaction with different azides to afford 1,2,3-triazole/thiadiazole hybrids **3a–c**. The structure of the synthesized compounds was confirmed using different spectroscopic analysis such as FT-IR, ^1^H NMR,^13^C NMR and elemental analysis. Moreover, the synthesized compounds were prepared in nano scale via chitosan to enhance their solubility and compatibility, and their size was evaluated via transmission electron microscope (TEM). The formulated nanoparticles are found to be relatively stable with higher positive zeta potential 22.5–29.5 mV and particle size 29–80 nm. The studied compounds were further subjected to molecular docking in the active site of four particular proteins AChE, BuChE, LOX-5 and COX-2.The synthesized compounds and their nanoformulations were tested as anti-inflammatory and anti-Alzheimer as acetylcholinesterase inhibitors. The result revealed that nanoformulations **N-(3a–c)** exhibited superior inhibitory activity compared to their synthesized counterparts **3a–c**, demonstrating enhanced potency against AChE, BuChE, NO formation, iNOS, LOX-5, and RBC lysis. **N-3a** showed the strongest iNOS inhibition, while **N-3b** was the most effective BuChE inhibitor. Notably, all nanoformulations matched the reference drug in LOX-5 inhibition and outperformed diclofenac K in protecting against RBC lysis. These results highlight the potential of these hybrids as anti-inflammatory and anti-Alzheimer agents.

**Supplementary Information:**

The online version contains supplementary material available at 10.1038/s41598-025-26140-8.

## Introduction

The synthesis of nitrogen-heterocycles is considered as one of the most appealing research topics in pharmaceutical and medicinal chemistry^1^. Click chemistry is a modular synthetic method for the synthesis of new molecular entities^2^. Click reaction afford a long-lasting and biocompatible product used in medicinal chemistry^3^, bio-conjugation^4^, and polymer functionalization^5^. In recent years, 1,2,3-triazoles have been the most extensively studied of the various kinds of nitrogen heterocycles^6^. Synthetic 1,2,3-triazoles are being investigated extensively in several drugs, including antibacterial^7^, antifungal^8^, anticancer^9^, anti-HIV^10^, antitubercular^11^, anti-inflammatory^12^ and anti-Alzheimer^13^ as acetylcholinesterase inhibitors^14,15^.

Heterocyclic compounds have diverse pharmacological effects. Among them, thiadiazol a five-membered heterocyclic scaffold, plays a crucial role in both natural and pharmaceutical drugs ^16,17^. Thiadiazol moiety perform as a “hydrogen binding domain”, also sulfur atom enhances lipo-solubility, resulting in analogues with increased lipophilicity^18^. 1,3,4-Thiadiazole scaffolds being widely researched in multiple drugs, including antifungal^19^, antibacterial^20,21^, anticancer^22^, anti-HIV^23^, antioxidant^24^, anti-acetylcholinesterase^25^, anti-inflammatory^26^, and carbonic anhydrase inhibitors^27^ such as acetazolamide and methazolamide.

Nanotechnology has drawn interest because of its ability to develop particles that are appealing to different cell kinds^28^. Compounds in nanoscale pose challenges for biological cells due to their enhanced reactivity and surface area^29^. Furthermore, nanoparticles provide superior enhanced characteristics compared to parent structures^30,31^. The aim of designing novel nanoparticle delivery systems is to enable regulated and precise release of pharmaceutical drugs as well as biodistribution^32^. The primary rationale for developing nanoformulations is to improve the bioavailability of poorly soluble drugs^33^.

Alzheimer’s disease (AD) is a multifactorial neurodegenerative disorder characterized by amyloid-β plaque accumulation, tau hyperphosphorylation, oxidative stress, and neuroinflammation. Recent studies have emphasized the importance of targeting both anti-inflammatory and anti-Alzheimer’s pathways, especially given the central role of neuroinflammation in the progression of Alzheimer’s disease. Activated microglia and astrocytes release pro-inflammatory cytokines that exacerbate neuronal damage and cognitive decline. Therefore, dual-targeting strategies are increasingly favored in drug design to address the multifactorial nature of neurodegenerative diseases^34,35^.

Therefore, the hybridization of 1,2,3-triazole and 1,3,4-thiadiazole scaffolds when formulated as nanoparticles could enhance the biological efficacy and provide dual-action compounds capable of modulating neuroinflammation and cholinergic dysfunction and improving bioavailability. This dual-target approach may offer a more effective therapeutic strategy for neurodegenerative conditions like Alzheimer’s disease. To test this hypothesis, our work aimed to synthesize new 1,2,3-triazole/thiadiazole hybrids via click reaction, then the synthesized hybrids **3a–c** were prepared in nano scale via chitosan ionic gelation method to afford encapsulated chitosan nano formula **N-(3a–c)**. The synthesized compounds and their nanoparticles were explored with their anti-inflammatory and anti-Alzheimer treatments based on experimental measurements and docking studies. Also, DFT calculations were performed in order to explore their potential thermodynamic stability, molecular geometry, frontier molecular orbitals energy gap and their molecular electrostatic potential map.

## Results and discussion

### Chemistry

**Scheme 1:**
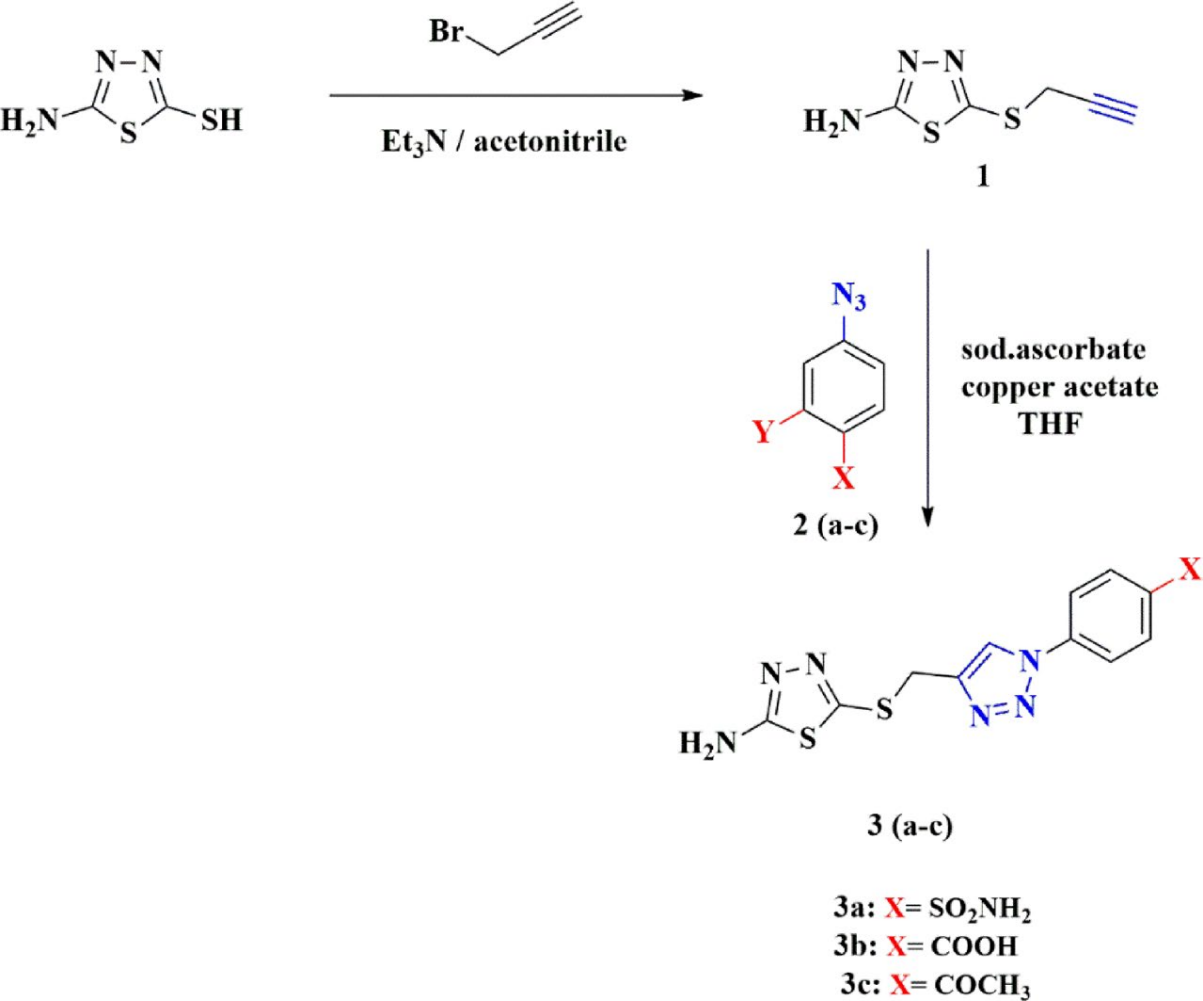
Synthesis of 1,2,3-triazole derivatives based on thiadiazols.

Herein, new 1,2,3-triazoles based on amino thiadiazols moiety via Click reaction were synthesized and characterized. As adopted in Scheme 1, selective alkylation of 5-amino-1,3,4-thiadiazol-2-thiol using propargyl bromide as alkylating agent in presence of triethylamine as a base was stirred in acetonitrile as solvent for 8 h. to afford 5-(prop-2-yn-1-ylthio)-1,3,4-thiadiazol-2-amine (**1**) in a high yield. Its structure was confirmed by IR spectroscopy, which showed a strong band at ν (cm^−1^): 3275 corresponds to the Csp-H stretch, and its ^1^H-NMR spectrum showed triplet peak at 6.34 ppm corresponds to the acetylenic bond C≡C**H**, and its ^13^C-NMR spectrum showed peaks at δ_C_: 85.2, 81.4 (C≡C).

A library of thiadiazol-triazole hybrid compounds **3a–c** were synthesized via click reaction of compound (**1**) with different azides namely 4-azido sulfanilamide, 4-azido benzoic acid or 4-azido acetophenone **2a–c**; respectively using a combination of copper acetate and sodium ascorbate. The structure of the synthesized compounds **3a–c** was confirmed using spectroscopic data. For compound **3b** its IR spectrum showed broad band of COOH at 3260 cm^−1^, and its ^1^H-NMR spectrum showed singlet peak of COO**H** at 13.22 ppm. After D₂O exchange spectra of **3b** shrink significantly of NH_2_ peak at 7.31 ppm and disappearing of OH peak. Moreover, ^1^H-NMR spectrum for compound **3c** showed a singlet peak of aliphatic CH_3_ at 2.57 ppm, and its ^13^C-NMR spectrum showed peaks at δc: 29.49 (CH_2_), 27.37 (CH_3_), as reported in Figure (S1-S14A) in the supporting information.

### Synthesized compounds in nm scale via chitosan nanoparticles.

**Fig. 1:**
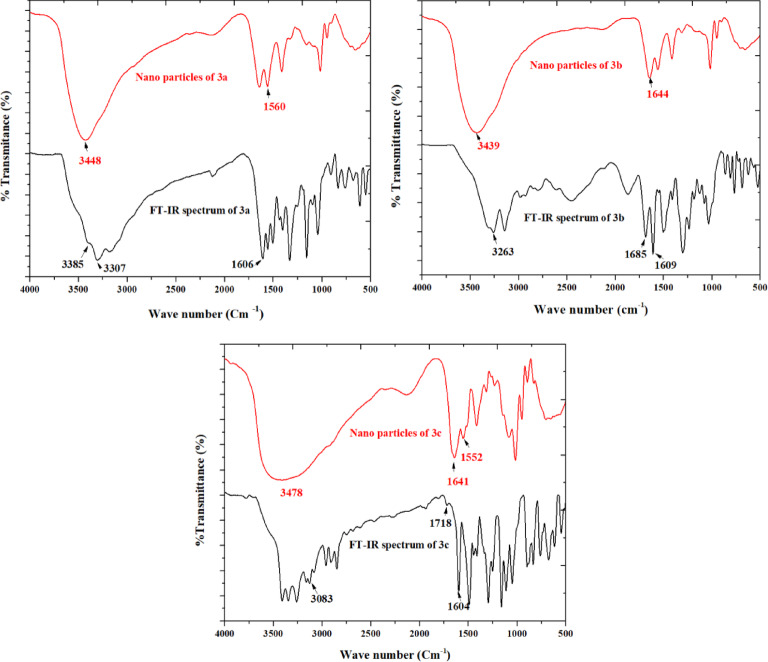
FT-IR of the synthesized compound **3a–c** and their nano-formulations.

**Fig. 2:**
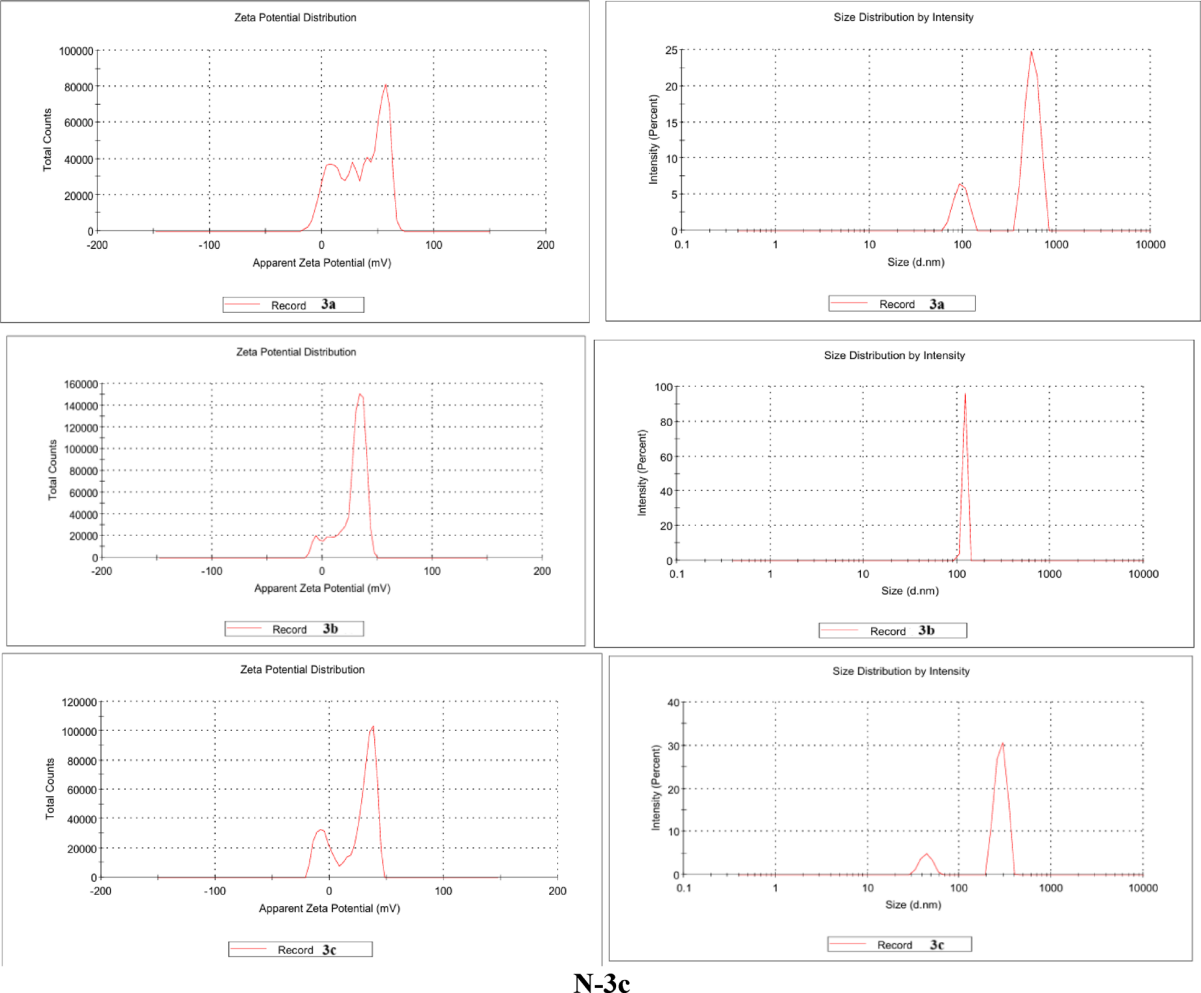
Dynamic light scattering** (**DLS) study of the synthesized nanoparticles showing zeta potential (left) and size distribution (right).

**Fig. 3:**
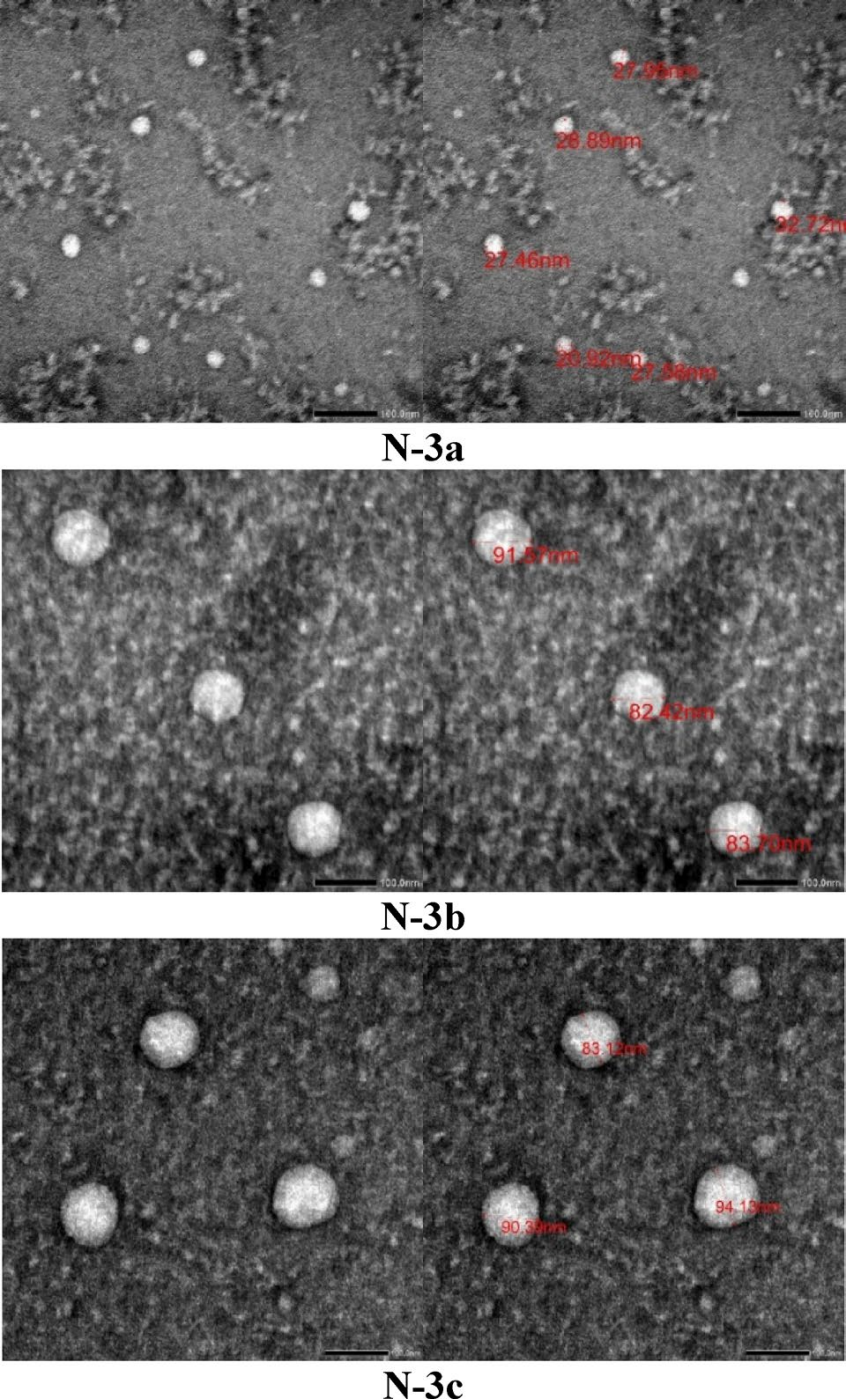
Transmission electron microscope study of the synthesized nanoparticles **N-(3a–c**).

The synthesized compounds **3a–c** on the nanometer scale via the ionic gelation method using chitosan nanoparticles were prepared. As shown in (Figs. 1, 2 and 3), the FT-IR of the synthesized compounds and their nano formulation establish an appropriate nanomaterial preparation. All nano formulations showed broad bands of chitosan about 3400–2980 cm^–1^. **N-3b** and **N-3c** showed strong band of **CO**OH and **CO**CH_3_ shifted to 1641 and 1685 cm^–1^ respectively. The formulated nanoparticles were relatively stable, with a positive net charge. **N-3a** showed the highest zeta potential of + 29.4 mV (Table 1).

**Table 1:** Physicochemical characteristics of the prepared nanoparticles.

Synthesized nanoparticles	Zeta potential (mv)	Average vesicle size (nm)	PDI
N-3a	29.4	29.2	0.994
N-3b	27.7	82.4	1.000
N-3c	22.4	83.1	0.931

### Biological evaluation

#### In silico physicochemical and pharmacokinetic property prediction

Swiss ADME was used to assess the physicochemical and pharmacokinetic characteristics of the promising compounds^36^. All three compounds **3a–c** showed complete compliance with both Lipinski’s and Ghose’s rules. Along with having zero Brenk or PAINS alert. As shown in (Table 2), additionally, both **3b** and **3c** showed bioavailability scores of 0.55. While **3a** and **3c** showed lead likeness. Finally, they had synthetic accessibility ranging from 3.21 to 3.05. The initial screening of the molecules ensured high drug likeness through complete compliance with both Lipinski’s and Ghose’s rules. Additionally, potential safety and exclusion of false positive results was evident through zero Brenk and PAINS alerts. Altogether with the three molecules easily synthesized, all three molecules are highly suitable candidates for further investigations. Furthermore, both **3b** and **3c** showed superior advantage of bioavailability, while **3a** and **3c** showed to have lead-likeness properties. Both advantages supported the candidacy of compounds for further testing.

**Table 2:** Showing drug-likness and lead-likness parameters of compounds calculated by SwissADME.

	3a	3b	3c
Molecular formula	C_12_H_10_N_6_O_2_S_2_	C_11_H_11_N_7_O_2_S_3_	C_13_H_12_N_6_OS_2_
Physicochemical properties			
Molecular weight	334.38	369.45	332.4
Canonical smiles	Nc1nnc(s1)SCc1nnn(c1)c1ccc(cc1)C(= O)O	Nc1nnc(s1)SCc1nnn(c1)c1ccc(cc1)S(= O)(= O)N	Nc1nnc(s1)SCc1nnn(c1)c1ccc(cc1)C(= O)C
Drug likeness			
Lipinski violations	0	0	0
Ghose violations	0	0	0
Bioavailability score	0.11	0.55	0.55
Pains alerts	0	0	0
Brenk alerts	0	0	0
Medicinal chemistry			
Lead likeness	Yes	No	Yes
Synthetic accessibility	3.05	3.21	3.07

#### Molecular docking and interactions with receptor amino acid residues

For initial screening as inhibitors of neuroinflammation, the three compounds **3a–c** were screened against AChE, BuChE, LOX-5 and COX-2 structures (PDB: 4EY7, 7Q1O, 6N2W and 5KIR, respectively). They showed docking scores between − 8.272 and − 3.036 with potential binding energies ranging between − 49.74 and − 2.58 (Table 3 and 4). Thus, 2D interactions with each target were examined for better understanding of potential mode of binding and mechanism of action. RMSD calculations for validation of protein preparation and generated grids upon docking of each crystal structure co-crystallized ligand showed values of 0.321, 1.010, 2.032 and 0.295 for 4EY7, 7Q1O, 6N2W and 5KIR, respectively. Indicating a high similarity to crystallographic pose and thus high reliability of subsequent docking results. AChE active site is composed of catalytic site triad, acyl pocket along with anionic site and oxyanion holes all of which are connected to the external environment by a tunnel^37^. TYR86, located at AChE catalytic anionic site (CAS) and essential residue for AChE binding affinity was a common target for all compounds, with **3b** targeting also PHE338 located at the same site^38,39^. All compounds-maintained Pi-Pi stack interaction with TRP286 of AChE, with both **3a** and **3c** forming hydrogen bond (H-bond) with TYR124, along with Pi-Pi stack interactions with TRP286. Additionally, **3a** had an extra H-bond formed with GLH202. While **3b** formed H-bonds with both GLH202 and TYR341, along with Pi-Pi stack interaction with PHE338 and TYR341. AChE and BuChE showing over 65% homology and having similar binding site structure of a gorge cavity^40^. The mode of binding showed to be similar, however, for BuChE all compound formed Pi-Pi stack interaction with catalytic aromatic/anionic site (CAS) residue TRP82, an essential catalytic residue. Furthermore, **3a** and **3b** form Pi-Pi interactions with PHE329 and TYR332, respectively. Altogether with, **3a-c** formed H-bonds with THR-120, GLH197 and TYR332, respectively. The low MMGBSA dG bind especially to 4EY7 can be attributed to low ligand efficiency of − 0.630 kcal/mol/Heavy Atom, describing how effectively each non-hydrogen atom is contributing to binding.

**Table 3:** Docking scores and estimated free binding energy (MMGBSA dG bind) of tested compounds against AChE, BuChE structures.

Compounds	AChE		BuChE	
	4EY7		7Q1O	
	Docking score	MMGBSA dG bind	Docking score	MMGBSA dG bind
3a	− 7.303	− 2.58	− 3.698	− 7.88
3b	− 7.508	− 26.94	− 4.966	− 26.92
3c	− 8.272	− 28.19	− 5.279	− 27.05

**Table 4:** Docking scores and estimated free binding energy (MMGBSA dG bind) of tested compounds against LOX-5, COX-2 structures.

Compounds	LOX-5		COX-2	
	6N2W		5KIR	
	Docking score	MMGBSA dG bind	Docking score	MMGBSA dG bind
3a	− 3.990	− 11.67	− 6.247	− 28.85
3b	− 3.036	− 19.98	− 6.374	− 49.78
3c	− 4.664	− 32.67	− 6.780	− 41.97

**Fig. 4:**
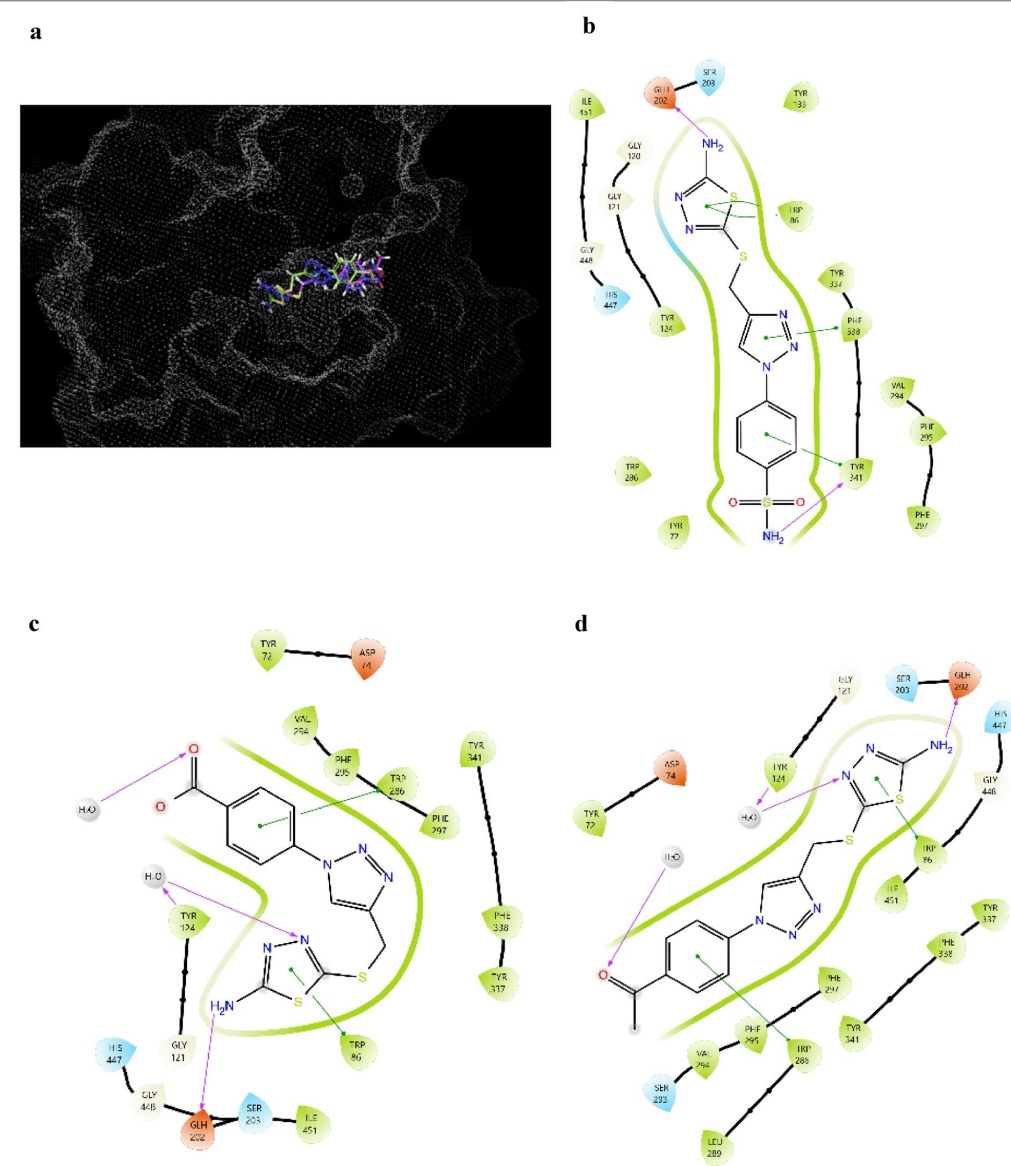

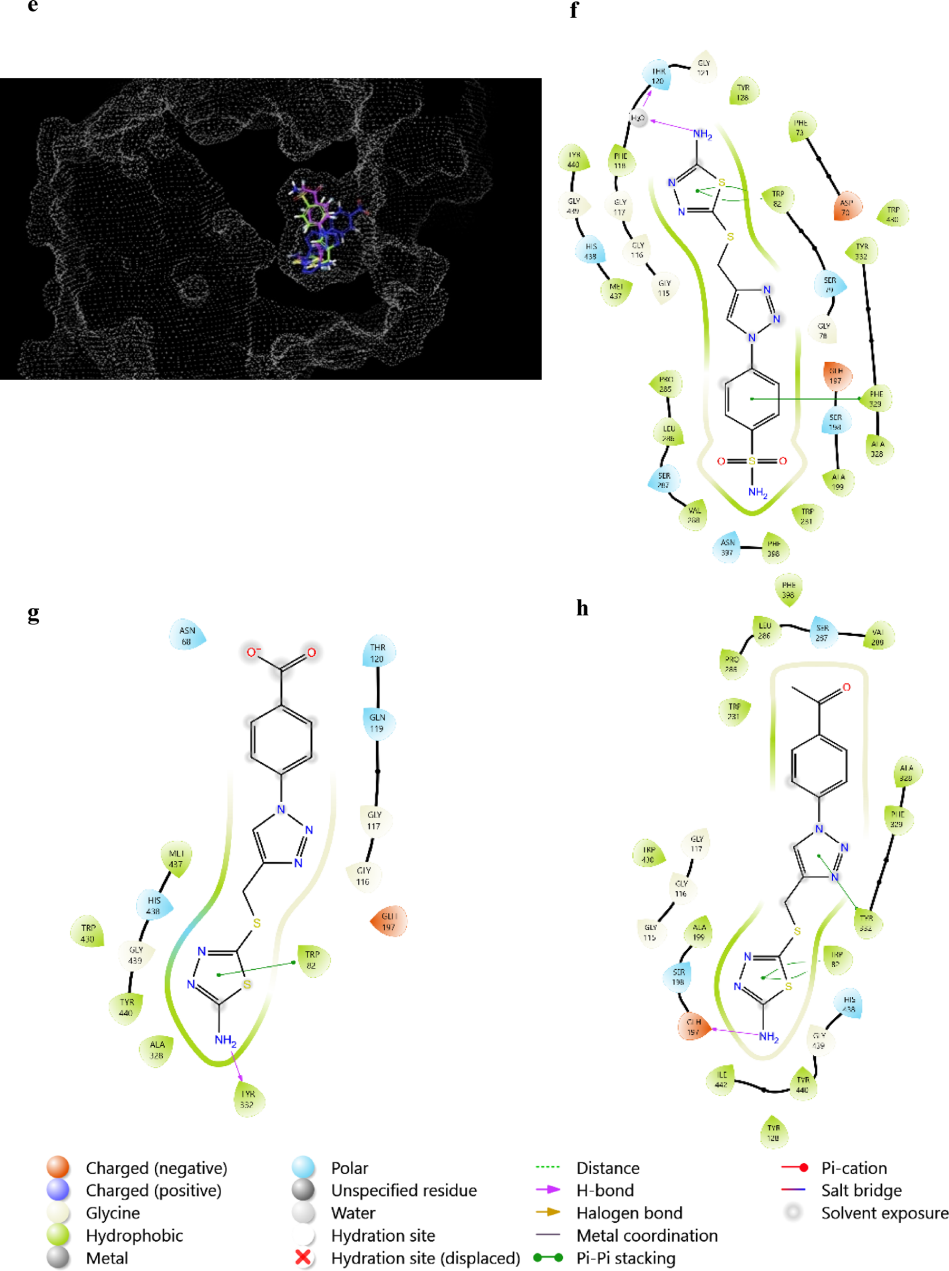
(**a** and **e**) show **3a** (blue), **3b** (green) and **3c** (purple) occupying the active site groove of AChE (4EY7) and BuChE (7Q1O), respectively. (**b**, **c** and **d**) show the binding mode of **3a**, **3b** and **3c** with 4EY7, respectively. (**f, g** and **h**) show the binding mode of **3a**, **3b** and **3c** with 7Q1O, respectively.

Altogether, considering both docking scores and binding mode of compounds with both cholinesterase, all compounds showed promising results during virtual screening as anti-cholinesterase, with abilities to occupy the active site groove of both enzymes (Fig. 4). More specifically **3b** showed a similar binding mode to BuChE to that of the FDA approved cholinesterase inhibitor galantamine rendering it a highly potential BuChE inhibitor^41^.

**Fig. 5:**
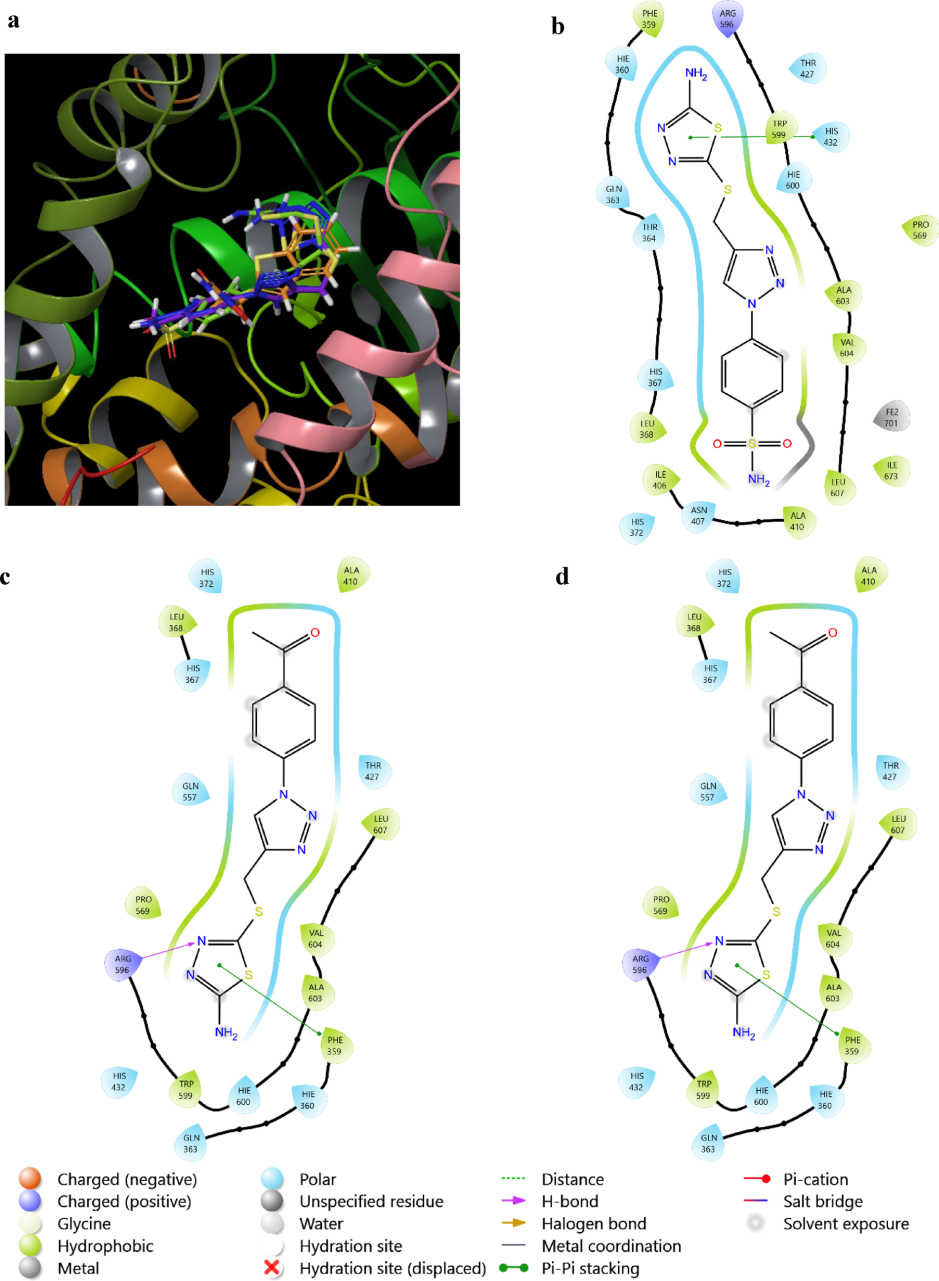
(**a**) shows **3a** (blue), **3b** (green) and **3c** (purple) occupying the same site as Zileuton (orange) in LOX-5 (6N2W). (**b, c** and **d**) show the binding mode of **3a**, **3b** and **3c** with 62NW, respectively.

Arachidonic acid inflammatory mediators are closely related to neurological diseases and inflammation. With LOX-5 and COX-2 being the main across those mediators, the ability of tested compounds to inhibit them was screened. In the case of LOX-5 both **3a** and **3c** could directly target the active site through interactions with residues ARG596 and PHE359. While **3b** targeted an active site related residue HIS432 through Pi–Pi interaction as shown in Fig. 5.

**Fig. 6:**
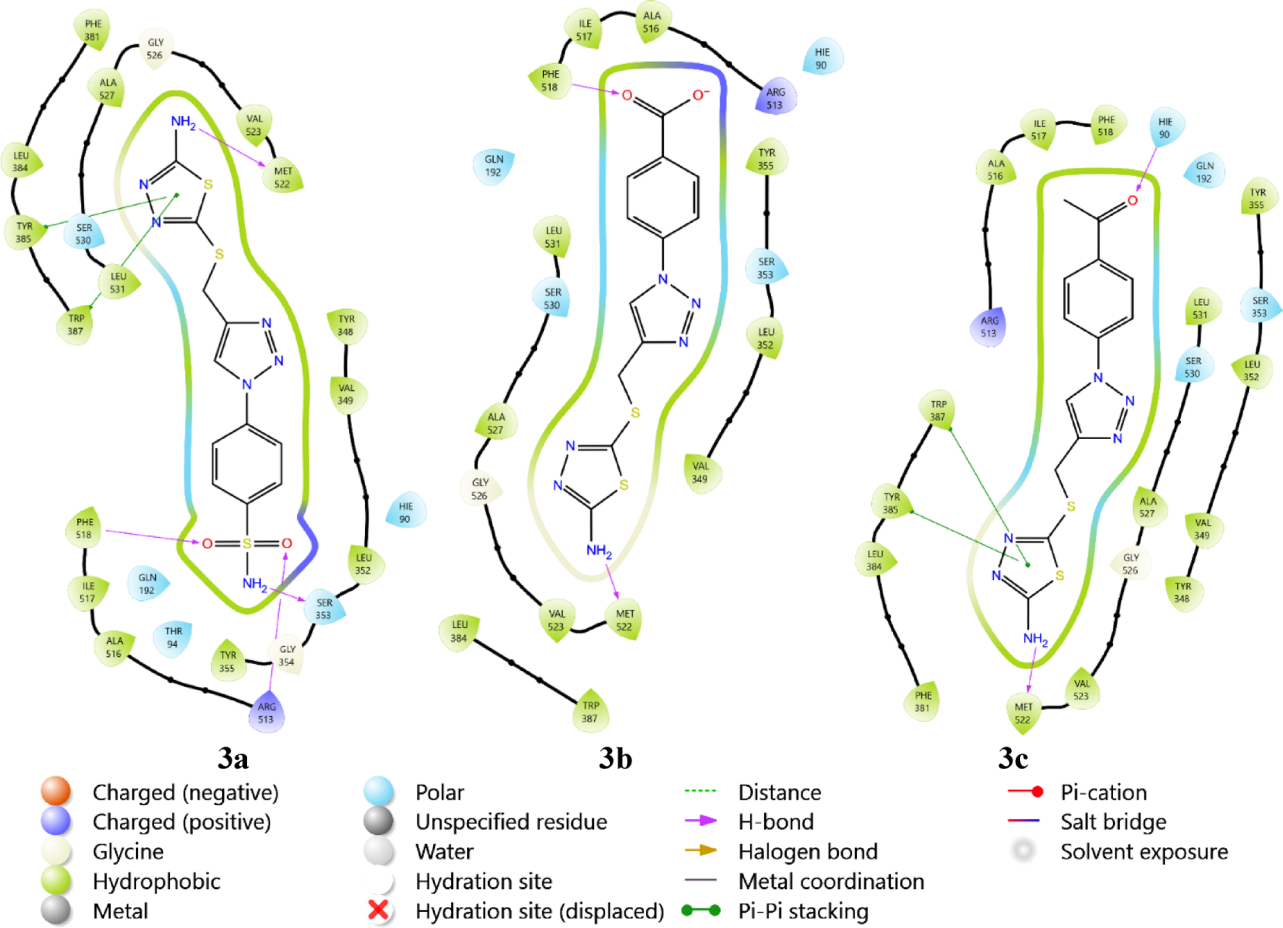
The binding mode of **3a**–**c** with COX-2 (5KIR).

On the other hand, the hydrophobic region of the active site MET522 of COX-2 was targeted by all tested compounds through formation of H-bonds. **3b** showed superior binding to the same site by targeting TRP387, TYR385 and PHE518, while **3a** and **3c** targeted only TRP387 & TYR385 and PHE518, respectively. **3b** could also bind and target ARG513 and SER353, while **3c** formed H-bond with HIE90 allowing both compounds to occupy the side pocket located at the active site region, as shown in Fig. 6. Thus, all three compounds showed promising binding mode targeting active site regions of both arachidonic acid inflammatory mediators. Highlighting them as potential anti-inflammatory with an emphasis on potentiality as anti-neuroinflammatory compounds; with **3b** showing superior binding mode against COX-2.

#### Inhibition of AChE and BuChE

**Fig. 7:**
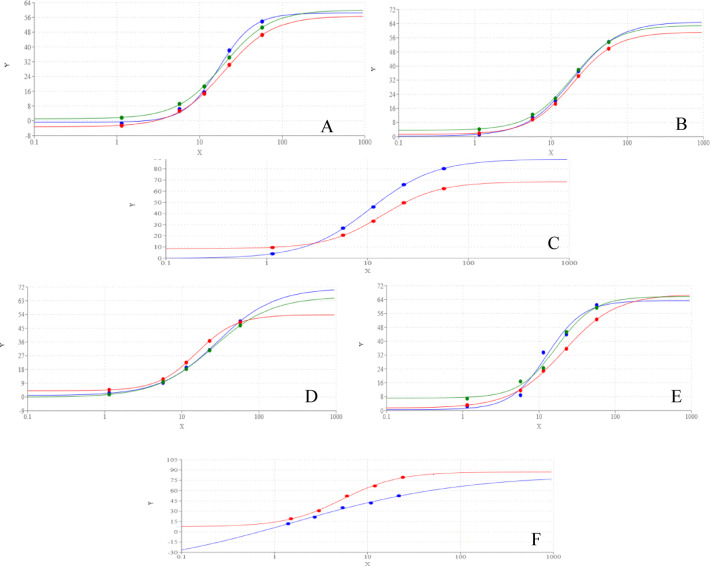
Dose response curve of different concentration of compounds **3**(green), **3b** (blue), **3c** (red), on AChE (**A**) and BuChe (**D**) and **N-3a**(green), **N-3b** (blue), **N-3c** (red) on AChE (**B**) and BuChe (**E**) and Donepezil HCl (blue) and Rivastigmine (red) on AChE (**C**) and BuChe (**F**).

Compounds **3a-c**, and their nanoparticle formulations **N-(3a–c)** exhibited concentration-dependent inhibition of AChE and BuChE (Fig. 7). The inhibitory potency was evaluated based on IC_50_ values, where lower IC_50_ indicates higher efficacy. For AChE inhibition, compounds **3b**, **N-3b**, and **N-3a** displayed the strongest activity, sharing the lowest IC_50_ values. **3a** and **N-3c** exhibited intermediate potency, while **3c** had the highest IC_50_, indicating the weakest inhibition. In contrast, for BuChE inhibition, **N-3b** was the most potent, followed by **N-3a**, **N-3c**, **3b**, **3c**, and finally **3a**, which showed the least inhibitory effect.

Notably, all tested compounds and their nanoformulations demonstrated moderate AChE and BuChE inhibitory activity compared to the reference drugs, as their IC_50_ values remained higher than those of standard inhibitors. However, nanoformulations exhibited significantly improved potency over their respective free compounds, emphasizing the enhanced bioactivity conferred by nanoparticle formulation.

#### NO scavenging and iNOS inhibition

**Fig. 8:**
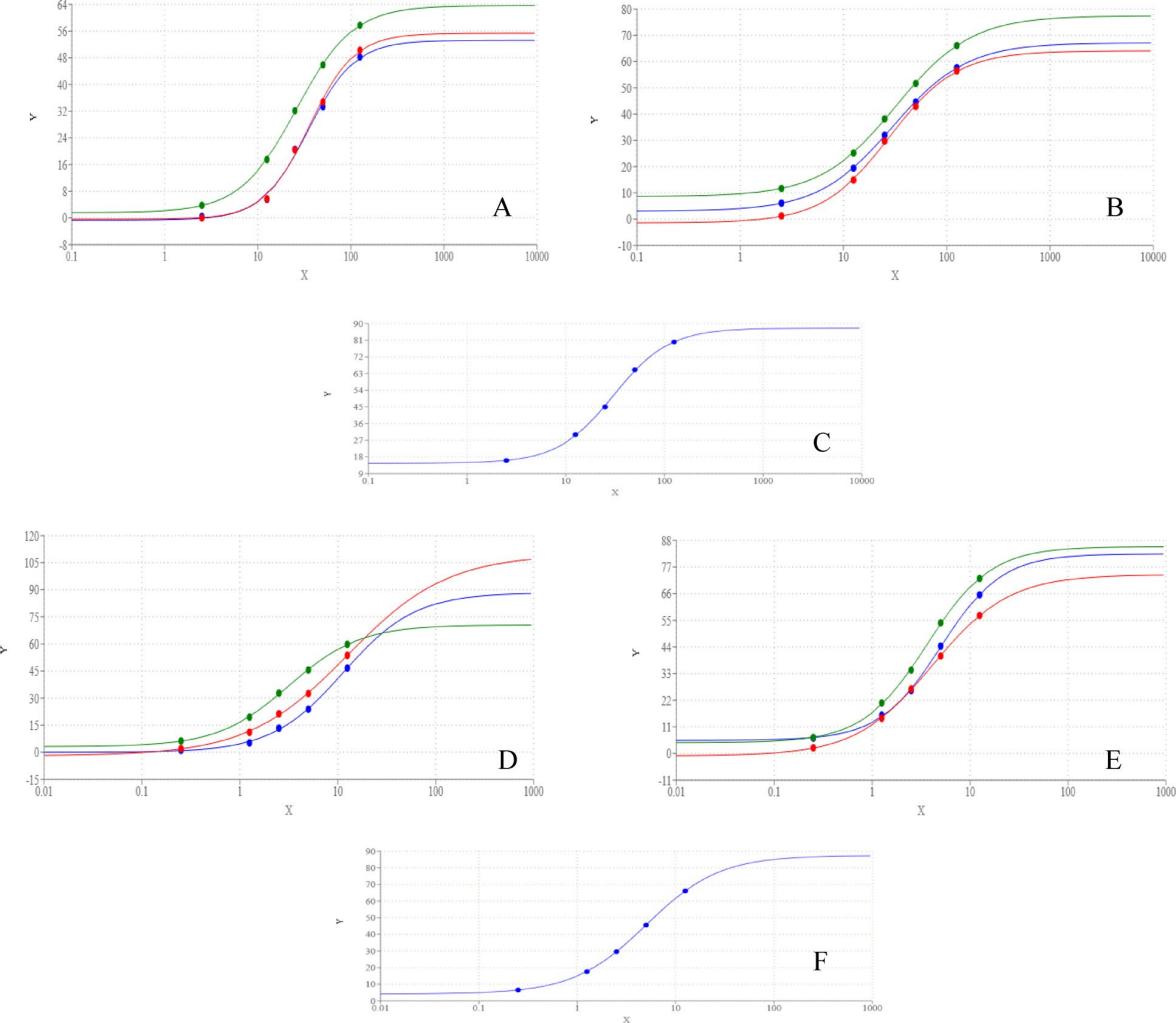
Dose response curve of different concentration of compounds **3a** (green), **3b** (blue), **3c** (red), on NO (**A**) and iNOS (**D**) and **N-3a** (green), **N-3b** (blue), **N-3c** (red) on NO (**B**) and iNOS (**E**), Ascorbic acid (blue) on NO (**C**) and **S**-Ethylisothiourea (blue) on iNOS (**F**).

All tested compounds and nanoformulations exhibited moderate NO scavenging activity in a concentration-dependent manner (Fig. 8). Among them, **N-3a** showed the strongest inhibition, followed by **N-3b** and **3a**, which had comparable IC_50_ values. **N-3c** demonstrated intermediate activity, while **3c** and **3b** were the least effective NO scavengers.

Regarding iNOS inhibition, **N-3a** displayed the lowest IC_50_, surpassing even the reference inhibitor, S-ethylisothiourea, indicating superior potency. **3a** and **N-3b** followed with equivalent IC_50_ values, while **N-3c**, **3c**, and **3a** exhibited progressively weaker inhibition. These findings suggest that nanoparticle formulations, particularly **N-3a**, significantly enhance iNOS inhibitory potential, which may contribute to anti-inflammatory effects.

#### LOX-5 inhibition

**Fig. 9:**
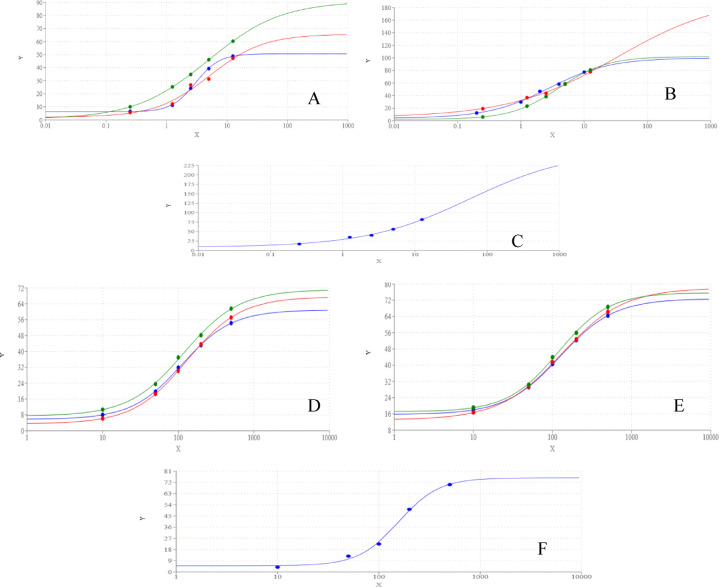
Dose response curve of different concentration of compounds **3a** (green), **3b** (blue), **3c** (red) on LOX5 (**A**) and RBC lysis (**D**) and **N-3a** (green), **N-3b** (blue), **N-3c** (red) on LOX5 (**B**) and RBC lysis (**E**), Zileuton (blue) on LOX5 (**C**) and diclofenac-K (blue) on RBC lysis (**F**).

All nanoformulations **N-(3a–c)** demonstrated LOX-5 inhibitory activity comparable to that of the reference drug (Fig. 9). In contrast, their corresponding synthesized compounds exhibited substantially weaker inhibition, with IC_50_ values ranging from twofold (**3a)** to 5.5-fold (**3b)** higher than the reference drug. These results highlight the superior pharmacological efficiency of nanoformulated compounds in targeting LOX-5.

#### RBC hemolysis inhibition

The ability of the tested compounds and their nanoformulations to prevent RBC lysis was assessed and compared to diclofenac K as a standard anti-inflammatory drug (Table 5). All nanoformulations exhibited lower IC_50_ values than diclofenac K, indicating greater protective effects against RBC lysis. Conversely, the synthesized compounds had higher IC_50_ values than diclofenac K, suggesting weaker membrane-stabilizing properties.

**Table 5:** IC50 of tested compounds on AChE, BuChE, LOX5, iNOS, NO and RBC lysis.

Compounds	AChE	BuChE	LOX5	NO	iNOS	RBC lysis
3a	57.61 ± 0.168d	71.11 ± 1.278h	6.37 ± 1.295b	63.08 ± 0.199c	6.28 ± 0.928c	213.96 ± 0.088e
3b	40.46 ± 0.051c	58.68 ± 0.832f.	20.52 ± 0.811d	171.24 ± 0.159f.	14.24 ± 0.774f.	322.98 ± 0.129g
3c	78.75 ± 0.125e	67.53 ± 0.856g	16.08 ± 0.847c	125.56 ± 0.204e	10.73 ± 0.849e	290.28 ± 0.074f.
N-3a	43.78 ± 0.069c	29.81 ± 0.794d	3.72 ± 0.842a	45.42 ± 0.166b	4.37 ± 0.602a	141.51 ± 0.261a
N-3b	44.31 ± 0.117c	25.95 ± 1.759c	2.56 ± 0.992a	68.25 ± 0.108c	6.28 ± 1.582c	172.04 ± 0.159c
N-3c	57.35 ± 0.063d	48.33 ± 0.637e	3.31 ± 0.669a	73.71 ± 0.192d	8.17 ± 0.706d	166.68 ± 0.207b
Reference drugs						
Donepezil HCl	12.87 ± 2.945a	18.37 ± 1.592b				
Rivastigmine	23.69 ± 1.742b	5.88 ± 1.855a				
Zileuton			3.74 ± 0.561a			
S-Ethylisothiourea					5.98 ± 0.737b	
Ascorbic acid				29.14 ± 0.973a		
diclofenac K						202.73 ± 1.673d

Overall, nanoparticle formulations consistently exhibited greater inhibitory activity than their corresponding synthesized compounds across all tested biological targets. The reduced IC_50_ values of nanoformulations indicate enhanced bioavailability and efficacy, potentially due to improved solubility, cellular uptake, and sustained drug release. Among the tested nanoformulations, **N-3b** and **N-3a** demonstrated the highest potency across multiple assays, particularly in LOX-5, iNOS and NO scavenging, reinforcing their potential as lead compounds for further drug development.

### DFT theoretical study

#### Geometrical structure

The synthesized compounds **3a-c** were theoretically investigated with DFT calculations. Gauss View 6.016^42^, to sketch compounds, calculated in the gas phase, and calculated by Gaussian 09 Revision D.01 software^43^, using DFT/B3LYP method at 6-311g basis sets.

**Fig. 10:**
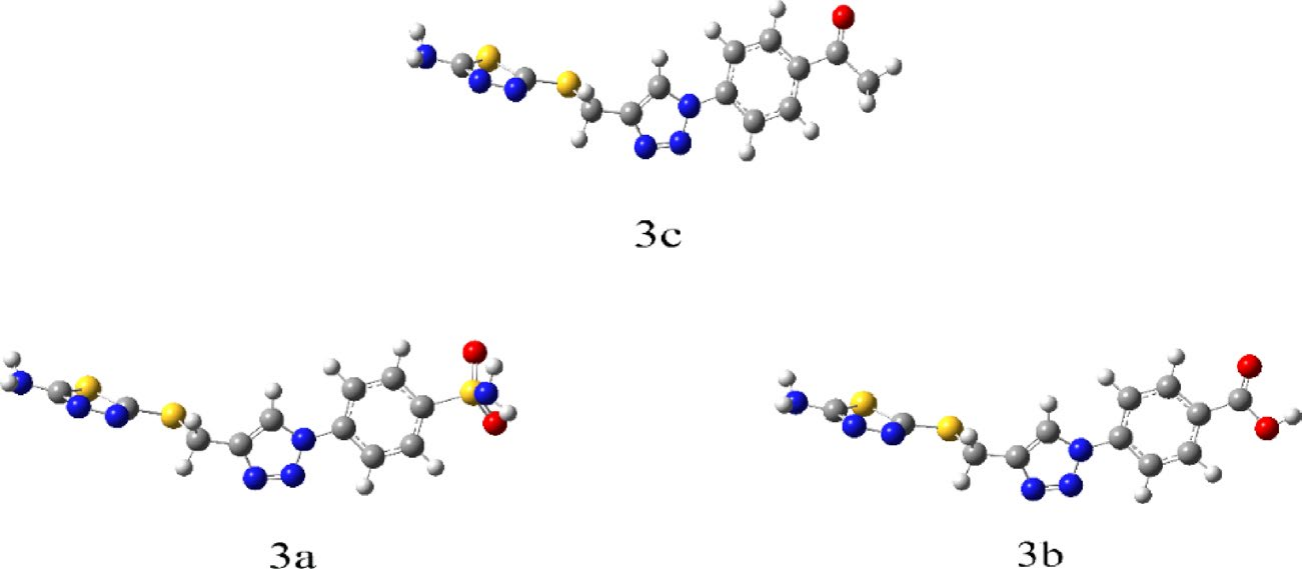
The Optimized geometrical structures of the synthesized thiadiazol derivatives compounds **3a–c** (grey color–Carbon, white color–Hydrogen, red color–Oxygen, and blue color–Nitrogen).

This approach made it easier to optimize molecular geometry in order to identify the structures with the lowest energy and highest stability, commonly referred to as convergence Furthermore, the optimized structures were subjected to a frequency procedure, with the same basis sets used to compute their thermodynamic characteristics. Additionally, the lack of imaginary frequencies indicated the stability of the optimized compounds. The optimized compounds are presented in (Fig. 10).

#### Polarizability & dipole moment

Molecular polarizability is the extent to which an external charge may distort a molecule’s electron cloud and cause it to acquire an electric dipole moment^44^. Terminal substituents’ electronic nature and polarity have a significant impact on the compound’s polarizability and dipole moment^45^. The estimated values of polarizability for the examined compounds are shown in (Table 6). Compound **3b** shows lower polarizability compared to the others owing to the presence of high electron withdrawing group (COOH) and carboxyl groups readily participate in hydrogen bonding, reduces the overall electron cloud flexibility, making it less polarizable.

**Table 6:** Calculated Polarizability **(**a.u**.)** and dipole moment (Debye) of compounds **3a–c.**

Compounds	Dipole moment				Polarizability
	X	Y	Z	Total	
3a	− 1.9268	4.1509	3.5043	5.7639	243.95
3b	− 0.5071	3.3946	0.6220	3.4881	230.53
3c	− 0.7523	2.3534	0.5289	2.5266	240.53

Assessment of dipole moments is conducted along the X, Y, and Z-axes. It is observed that compound **3a** possesses higher dipole moment due to presence of sulfonamide group then **3b** due to presence of COOH group.

#### Molecular electrostatic potentials (MEP)

Molecular electrostatic potential (MEP) is a useful instrument for determining a molecule’s charge distribution and electron density. Consequently, it facilitates the prediction of intramolecular and intermolecular interactions, the assessment of molecular packing, and the computation of atoms’ formal and partial charges^46^. Utilizing the same basis set, charge distribution maps were generated and displayed for each compound using the MEP investigation. The charge distribution map shows areas of electron density on molecules in ascending order: red > orange > yellow > green > blue. Therefore, areas with the least amount of negative charge are represented by blue, whereas areas that have significant electronegativity will be shown in red^47^.

**Fig. 11:**
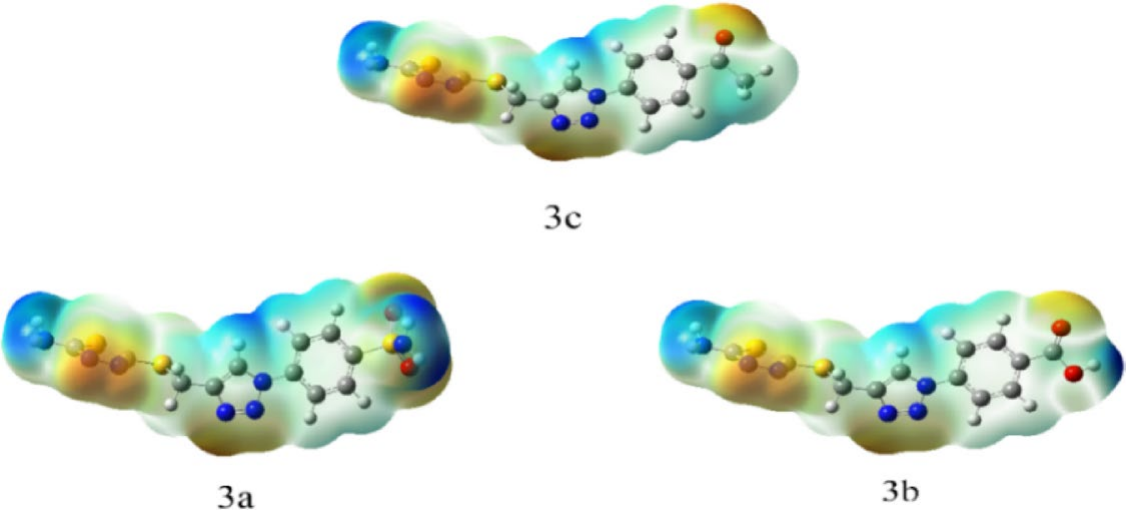
Molecular Electrostatic Potential (MEP) Maps of the synthesized compounds.

It is noted that the presence of the thiadiazol group, which gives each sulfur atom a half-negative charge, results in a larger negative charge on the terminal of all prepared compounds **3a–c**, while for compound **3a** high negative charge is located on the oxygen atom of sulfonamide group. MEPs maps are shown in (Fig. 11).

#### Frontier molecular orbitals (FMOs)

**Fig. 12:**
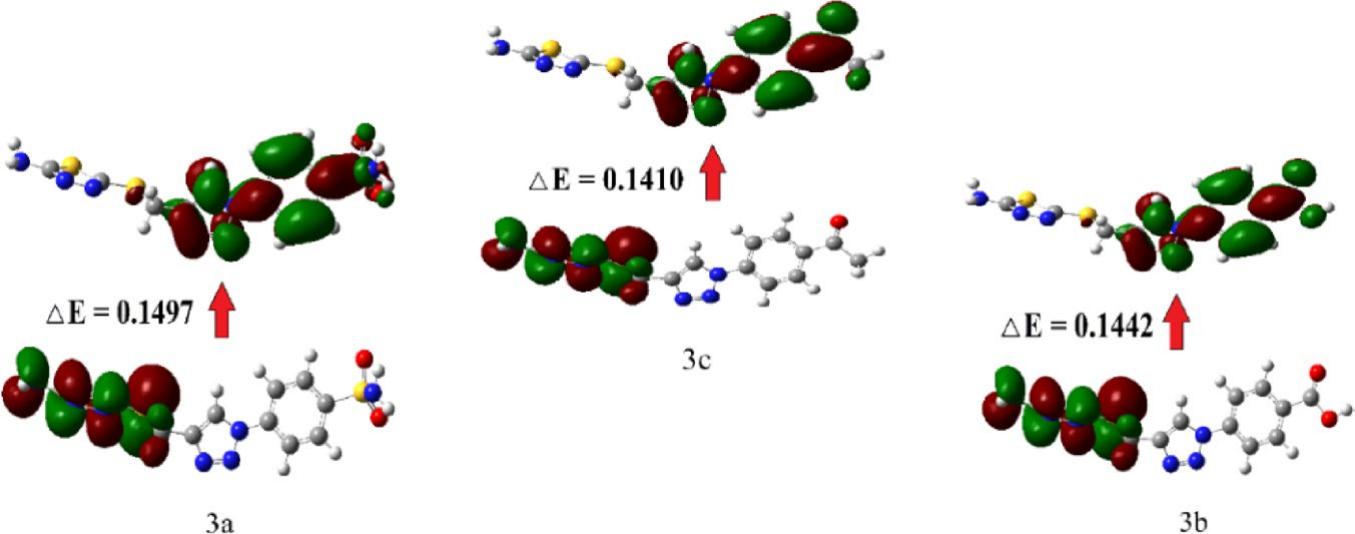
Graphical representation of the frontier molecular orbitals (FMOs) of compounds **3a–c**.

Frontier molecular orbitals (FMOs) are the lowest unoccupied molecular orbital (LUMO), that receives electrons, and the highest occupied molecular orbital (HOMO), that donates electrons. These parameters are helpful for investigating molecular reactivity because they anticipate electron transport and excitation processes between orbitals, as demonstrated by the energy gap between HOMO and LUMO. Excitation energy increases and reactivity decreases as the energy gap rises. Reactivity rises when the energy gap is smaller because excitation energy falls^48^. They are presented graphically in (Fig. 12). The results of FMO reactivity descriptors are presented in (Table 7). Compounds’ biological activity is affected by their redox state, which in turn influences their electronic chemical potential. Compounds with a high electronic chemical potential, for instance, could serve as powerful oxidizing agents while also playing a role in redox processes necessary for signaling and cellular metabolism^49^. As a result, compound **4c** shows greater biological activity owing to its higher electronic chemical potential.

**Table 7:** The FMOs energies (eV), their energy difference (eV), and their corresponding parameters of the investigated compounds.

Compounds	E_HOMO_	E_LUMO_	ΔE	η	S = 1/η	X	μ =—X	∆N = −μ/η
3a	− 0.2265	− 0.0768	0.1497	0.0748	13.3636	0.1516	− 0.1516	2.0265
3b	− 0.2250	− 0.0808	0.1442	0.0721	13.8667	0.1529	− 0.1529	2.1204
3c	− 0.2250	− 0.0840	0.1410	0.0705	14.1864	0.1545	− 0.1545	2.1915

Softness refers to a substance’s π electron cloud’s susceptibility to disruptions caused by chemical processes. As a result, the electrical properties of terminal substituents may have an effect on softness^50^. As a result, all compounds **3a–c** have higher softness values due to the presence of the thiadiazol group.

## Materials and methods

### Materials and equipment

The material and equipment characterization are reported in the supporting information.

### Chemistry

#### 5-(prop-2-yn-1-ylthio)-1,3,4-thiadiazol-2-amine (1).

A mixture of 5-amino-1,3,4-thiadiazole-2-thiol (0.01 mol) and propargyl bromide (0.013 mol) in presence of triethylamine (0.02 mol) was refluxed in 25 ml of acetonitrile for 8 h, the excess solvent was removed under reduced pressure then the residue was poured into cold water (200 ml). The obtained solid was filtered off and crystallized from ethanol to give compound **1** as orange crystals (81% yield); R_f_ = 0.45 ( n-Hexane : ethyl acetate, 1:2, V/V); m.p = 164–166 °C; IR(KBr) ν_max_ (cm^−1^): 3276, 3113 (NH_2_), 2938 (Csp^3^-H) stretch were observed as strong bands; ^1^H NMR (500 MHz, DMSO-d6) δH: 7.38 (s, 2H, **NH**_**2**_), 6.35 (t, *J* = 7.0 Hz, 1H, CH_2−_ C≡**CH**), 5.21 (d, *J* = 6.5 Hz, 2H, **CH**_**2**−_ C≡CH); ^13^C NMR (125 MHz, DMSO-d6) δC: 172.6, 171.0 (thiadiazol), 85.2, 81.4 (C≡**CH** ), 46.1 (**CH**_**2**_); Anal. calculated for C_5_H_5_N_3_S_2_ (171.24): C, 35.07; H, 2.94; N, 24.54; Found: C, 35.15; H, 3.07; N, 24.68.

#### General procedure for click reaction

A mixture of 5-(prop-2-yn-1-ylthio)-1,3,4-thiadiazol-2-amine (**1**)(0.01 mol) and the appropriate azido aromatic compounds **2a–c** (0.011 mol): 4-azidosulfanilamide, 4-azidobenzoic acid, 4-azidoacetophenone in 15 ml THF, then add sodium ascorbate (0.004 mol) and copper acetate (0.002 mol) dissolve in distilled water (1ml) to the reaction mixture. The reaction mixture was stirred for 4–6 h, and the reaction progress was monitored with TLC. After the reaction completion, the reaction mixture was poured on cold water (100 ml), the obtained products were washed with water and dried to afford the desired products.

**3.2.3.**4-(4-(((5-Amino-1,3,4-thiadiazol-2-yl)thio)methyl)-1*H*-1,2,3-triazol-1-yl)benzenesulfonamide (**3a**).

Compound **3a** as yellowish-white crystals (82% yield); R_f_ = 0.55 ( n-Hexane : ethyl acetate, 1:2, V/V); m.p = 232–234 °C;IR(KBr) ν_max_ (cm^−1^): 3307, 3385(NH_2_), 1606(C = N) were observed as strong bands; ^1^H NMR (500 MHz, DMSO-d6) δH: 8.88 (s, 1H, **CH-**triazole), 8.41–8.09 (m, 2H, Ar–H), 7.98–7.78 (m, 2H, Ar–H), 7.49 (s, 2H, **NH**_**2**_), 7.31 (s, 2H, SO_2_**NH**_**2**_), 5.42 (s, 2H, **CH**_**2**_); ^13^C NMR (125 MHz, DMSO-d6) δC: 159.1, 144.5, 138.9, 128.0, 123.1, 120.9 (Ar–C), 56.6, 29.5 (CH_2_); Anal. calculated for C_11_H_11_N_7_O_2_S_3_(369.45): C, 35.76; H, 3.00; N, 26.54; Found: C, 35.83; H, 3.09; N, 26.63.

#### 4-(4-(((5-Amino-1,3,4-thiadiazol-2-yl)thio)methyl)-1*H*-1,2,3-triazol-1-yl)benzoic acid (3b)

Compound **3b** as pale yellow crystals (85% yield); R_f_ = 0.43 ( n-Hexane : ethyl acetate, 1:2, V/V); m.p = 228–230 °C; IR(KBr) ν_max_ (cm^−1^): 3260 (OH) broad band, 1686 (COOH), 1610(C = N); ^1^H NMR (500 MHz, DMSO-d6) δH: 13.23 (s, 1H, **COOH**), 8.79 (s, 1H, **CH-**triazole), 8.07 (d, *J* = 8.5 Hz, 2H, Ar–H), 7.98 (d, *J* = 8.0 Hz, 2H, Ar–H), 7.30 (s, 2H, **NH**_**2**_), 4.39 (s, 2H, **CH**_**2**_); D_2_O exchange-^1^H NMR (500 MHz, DMSO-d6) δH: 8.80 (s, 1H, **CH-**triazole), 8.07 (d, *J* = 8.5 Hz, 2H, Ar–H), 7.98 (d, *J* = 8.0 Hz, 2H, Ar–H), 4.41 (s, 2H, **CH**_**2**_); ^13^C NMR (125 MHz, DMSO-d6) δC: 166.9 (C = O), 145.1, 139.9, 131.7, 130.6, 122.5, 120.3 (Ar–C), 29.5 (CH_2_); Anal. calculated for C_12_H_10_N_6_O_2_S_2_(334.38): C, 43.10; H, 3.01; N, 25.13; Found: C, 43.18; H, 3.07; N, 25.26.

#### 1-(4-(4-((5-Amino-1,3,4-thiadiazol-2-yl)thio)methyl)-1*H*-1,2,3-triazol-1-yl)phenyl)ethanone (3c)

Compound **3c** as off-white crystals (83% yield); R_f_ = 0.47 ( n-Hexane : ethyl acetate, 1:2, V/V); m.p = 225–227 °C; IR(KBr) ν_max_ (cm^−1^): 3083 (C-H) sp^3^stretch, 1718 (C = O, ketone) were observed as strong bands; ^1^H NMR (500 MHz, DMSO-d6) δH: 8.82 (s, 1H, **CH-**triazole), 8.10 (d, *J* = 8.5 Hz, 2H, Ar–H), 8.01 (d, *J* = 9.0 Hz, 2H, Ar–H), 7.31 (s, 2H, **NH**_**2**_), 4.40 (s, 2H, **CH**_**2**_), 2.58 (s, 3H, **CH**_**3**_); D_2_O exchange-^1^H NMR (500 MHz, DMSO-d6) δH: 8.81 (s, 1H, **CH-**triazole), 8.10 (d, *J* = 8.5 Hz, 2H, Ar–H), 8.01 (d, *J* = 9.0 Hz, 2H, Ar–H), 4.42 (s, 2H, **CH**_**2**_), 2.59 (s, 3H, **CH**_**3**_); ^13^C NMR (125 MHz, DMSO-d6) δC: 197.5 (C = O), 145.1, 139.9, 136.8, 130.6, 122.5, 120.2 (Ar–C), 29.5 (CH_2_), 27.4 (CH_3_); Anal. calculated for C_13_H_12_N_6_OS_2_(332.40): C, 46.97; H, 3.64; N, 25.28; Found: C, 47.01; H, 3.69; N, 25.32.

### Nanoparticles synthesis and characterization.

A 100 mL solution of acetic acid (2% v/v) was mixed with 0.5 g of chitosan (100–150 kDa, DDa 85%, Sigma-Aldrich, Saint Louis, MO, USA), agitated for 30 min, and filtered using Whatman filter paper no1. The **N-3a**, **N-3b**, and **N-3c** solutions were added to the produced sodium tri-poly phosphate (TPP) solution in separate steps. Deionized water was used to make the solution (0.2% w/v). After the chitosan solution was made, each component was added dropwise while stirring constantly for 30 min. For future studies, the precipitate was kept in sterile falcon tubes at 4^◦^C^51^.

### Biological Evaluation

#### Inhibition activity of plant extract against acetylcholine esterase

In ELISA plate (Bio Tec. USA), 150 μl of phosphate buffer (0.1 M, pH 8) was directly added in ELISA blank well and 130 μl of phosphate buffer was added in ELISA activity wells. To the blank and activity wells, 5 μl of substrate ACTI (75 mM in distilled water ) was added, then 20 μl electric eel AChE + 20 μl of (of compound different concentration (test)/ DMSO (control) were added in activity ELISA wells only. The plate was preincubated for 15 min at 37 °C before the addition of the second substrate (DTNB). DTNB (60 μl, 0.32 mM in 10 ml phosphate buffer 0.1 M, pH 8) was added in both the blank and activity wells. Absorbance was measured at 405 nm every two min. Values obtained were analyzed and blank reading was subtracted from sample readings^52^.

Acetylcholine esterase inhibition activity was estimated from the following formula: [(A_C_–A_t_)/A_C_] × 100, where Ac represents the reaction rate without inhibitors, and At represents the reaction rate in the presence of inhibitors. The IC₅₀ value for each sample compound was determined using a nonlinear variable slope of the log (inhibitor) vs. normalized response curve.

#### Determination of nitric oxide scavenging activity

This method consisted of the addition of 50 µl of the same serial concentrations of compounds **3a–c** (10, 50, 100, 200 and 500 µg/ml), distilled water (as negative control) with 50 µl of 10 mM sodium nitroprusside solution (10 mM in distilled water ) into a 96-well plate. The plate was incubated under light at room temperature for 90 min. Finally, an equal volume of Griess reagent (1% of sulphanilamide and 0.1% of naphthylethylenediamine in 2.5% H_3_PO_4_) was added to each well to measure the nitrite content immediately at 490 nm using optima spectrophotometer. The test carried out triplicate^53,54^.

The Percentage of NO scavenging activity was calculated by using this equation [(A_C_–A_t_)/A_C_] × 100, Where; A_C_: The mean of absorbances of negative control_,_ A_t_: The mean of absorbances of test compound.

#### In vitro determination of anti-inflammatory effect of compounds using human red blood corpuscles membrane stabilizing method

A number of three rabbits, that were provided from Animal facility of Center of Excellence for Drug Preclinical Studies (CE-DPS), Pharmaceutical and Fermentation Industry Development Center, City of Scientific Research & Technological Applications (SRTA-city), were used in this study. The experimental design was approved by the committee applications for the institutional animal care and use committees (IACUC- SRTA city with approval number Tx-Rb-17–3-9–2025). The rabbit blood samples (animal facility of Center of Excellence for Drug Preclinical Studies (CE-DPS), Pharmaceutical and Fermentation Industry Development Center, City of Scientific Research & Technological Applications (SRTA-city)) were collected in heparinized tubes and washed three times with isotonic buffered solution (154 mM NaCl) in 10 mM sodium phosphate buffer (pH 7.4)) through centrifugation each time for 10 min at 3000 xg. Membrane stabilizing activity of the extract was assessed using hypotonic solution (((50 mM NaCl) in 10 mM sodium phosphate buffer saline (pH 7.4)) -induced human erythrocyte hemolysis. The test sample consisted of stock erythrocyte (RBC) suspension (0.5 ml) mixed with 5 ml of hypotonic solution containing serial concentration of the extracts (10, 50, 100 ,200, 500 ug/ml). The control sample consisted of 0.5 ml of RBCs mixed with hypotonic-buffered saline solution alone. The mixtures were incubated for 10 min at room temperature and centrifuged for 10 min at 3000 × g and the absorbance of the supernatant was measured at 540 nm using optima spectrophotometer. The test carried out triplicate^55^.

% Inhibition of hemolysis was calculated by {A_C_–A_t_/A_C_} × 100, Where; A_C_ = the mean of absorbances of hypotonic-buffered saline solution alone, A_t_ = the mean of absorbances of tested compound in hypotonic solution.

Inhibition of hemolysis activity of plant extract was expressed as IC50. IC50 value (mg/ml) is the inhibitory concentration at which 50% of hemolysis are repressed.

#### 5-Lipoxygenase (LOX-5) spectrophotometric assay

The LOX-5 activity assay was performed spectrophotometrically by monitoring the formation of hydroperoxyeicosatetraenoic acid (HPETE) at 234 nm, which reflects the oxidation of arachidonic acid by the enzyme. The reaction was conducted in a 96-well plate containing 180 μL of 50 mM phosphate buffer (pH 7.4), 10 μL of the test compound (dissolved in DMSO or ethanol), and 10 μL of purified LOX-5 enzyme (final activity: 75 U/mL). The reaction mixture was pre-incubated at 25°C for 5 min, followed by the addition of 10 μL of 50 μM arachidonic acid to initiate the reaction. Absorbance was recorded immediately at 234 nm using a UV–Vis spectrophotometer or a microplate reader, with continuous monitoring for 3–5 min at 30-s intervals^56^.

The percentage inhibition of LOX-5 activity was calculated using the formula: [(AC–At)/AC] × 100, where Ac represents the reaction rate without inhibitors, and at represents the reaction rate in the presence of inhibitors. The IC₅₀ values were determined by nonlinear regression analysis of a dose–response curve. A known LOX-5 inhibitor (e.g., Zileuton) was used as a positive control, while a negative control (reaction without an inhibitor) was included to establish 100% enzyme activity. Each experiment was conducted in triplicate to ensure accuracy and reproducibility.

#### The inhibitory activity of compounds to human iNOS by in vitro study

The assay was conducted following the procedure outlined in the iNOS Inhibitory Screening Kit (Catalogue No. EINO-100; EnzyChrom-BioAssay). Various concentrations of each compound and S-Ethylisothiourea (selective inhibitor of inducible nitric oxide synthase (iNOS)) were prepared in the following range (2.5, 5.0, 7.5, 10.0, and 12.5 µg/mL) was used as the reference. The enzyme solution (50 Unit, 100 µL) was dissolved in dH2O (3.9 mL), resulting in a 12.5 U/mL iNOS solution. A volume of 10 µL of enzyme solution, 25 µL of assay buffer, and 5 µL of test samples were injected into a 96-well plate and pre-incubated at 22 ± 2 °C for 15 min. The plate was then incubated at 37 °C for 60 min. A 200 µL quantity of NO detection reagent was added, and the detection reaction was carried out at 37 °C for 60 min. Absorbance was measured at a wavelength of 500–570 nm using an ELISA plate reader (ThermoFisher Scientific) at 540 nm. The average of five readings was used to calculate the percentage of iNOS inhibition using the following formula: ([(A_C_–A_t_)/A_C_] × 100, where At refers to the absorbance value of a test compound subtracted by the absorbance value of the blank well (without substrate) at 60 min, and Ac represents the absorbance value of the control subtracted by the absorbance value of the blank well (without substrate) at 60 min. The IC50 values and standard deviations were determined from the nonlinear regression dose–response curve^57^.

#### Butyrylcholinesterase (BuChE) activity

The assay was performed by initially incubating 10 μL of reference drugs and designed compounds with 50 μL of hBChE (final concentration: 0.06 U/mL) for 30 min. Following the incubation, 30 μL of 15 mM BTCI was added and further incubated for another 30 min. Subsequently, 160 μL of 1.5 mM DTNB was introduced into the respective wells, and absorbance was immediately measured at λ = 412 nm using a Multimode Microplate Reader (BioTek Synergy, USA). Blank readings were recorded for all compounds except for the enzyme, accounting for the non-enzymatic hydrolysis of the substrates^58^.

The reaction rate was compared in the presence and absence of inhibitors, and the percentage of inhibition was calculated using the formula: [(A_C_–A_t_)/A_C_] × 100.

where Ac represents the reaction rate without inhibitors, and At represents the reaction rate in the presence of inhibitors. The IC₅₀ value for each sample compound was determined using a nonlinear variable slope of the log (inhibitor) vs. normalized response curve.

The assay was conducted in three independent experiments, each performed in triplicate, to ensure the validity of the experimental results.

## Conclusion

This study portrays the design and synthesis of new 1,2,3-Triazole/Thiadiazole Hybrids **3a–c**, then synthesized in nanoscale with chitosan for targeting anti-inflammatory and anti-Alzheimer. The results revealed that all synthesized compounds demonstrated greater inhibitory activity. Moreover, the screened compounds were further subjected to molecular docking in the active site of AChE, BuChE, LOX-5 and COX-2 structures, Lipinski’s rule, and Swiss ADME filter. Particularly the nano formulations **N-(3a–c)** exhibit superior inhibitory activity compared to their synthesized counterparts**(3a–c)**, demonstrating enhanced potency against AChE, BuChE, NO formation, iNOS, LOX-5, and RBC lysis. **N-3a** showed the strongest iNOS inhibition, while **N-3b** was the most effective BuChE inhibitor. Notably, all nanoformulations matched the reference drug in LOX-5 inhibition and outperformed diclofenac K in protecting against RBC lysis. Then, we present density functional theory (DFT) calculations on the synthesized compounds **3a–c** to explore their potential thermodynamic stability, molecular geometry, frontier molecular orbitals energy gap investigation, as well as their molecular electrostatic potential mapping. These results highlight the potential of nanoformulations for developing more effective neuroprotective and anti-inflammatory therapeutics. Further studies are needed to assess their pharmacokinetics and in vivo efficacy.

## Supplementary Information

Below is the link to the electronic supplementary material.

Supplementary Material 1

## Data Availability

All data generated or analyzed during this study are included in this manuscript.
