# Supplementary material for: Design and synthesis of 1,2,3-triazole thiadiazole hybrids with in vitro and in silico evaluation of their anti-inflammatory and anti-alzheimer activities
Source: Sci Rep. 2025 Nov 21;15:41157. doi: 10.1038/s41598-025-26140-8 (PMC12638865; doi:10.1038/s41598-025-26140-8)
Supplement: Supplementary file 1 — Supplementary Material 1 [file 41598_2025_26140_MOESM1_ESM.pdf]

Supplementary information

Design and Synthesis of 1,2,3-Triazole Thiadiazole Hybrids with In Vitro and In Silico Evaluation of Their Anti-inflammatory and Anti-Alzheimer Activities

Ahmed R. Rabee [1*], Hamida Abdel-Hamid [1], Saied M. Soliman [1], Samah Ashraf [2], Ahmed A. Sobhy[2,3], Doaa Ahmad Ghareeb [2,3,4], Aalaa k. saad [1], Mohamed Hagar [1*]

[1] Chemistry Department, Faculty of Science, Alexandria University, P.O. Box 426, Alexandria, 21321, Egypt
[2] Bio-Screening and Preclinical Trial Lab, Biochemistry Department, Faculty of Science, Alexandria University, Alexandria, Egypt
[3] Microbiology and Immunology department, Faculty of pharmacy, Alexandria University, Alexandria, Egypt
[4] Center of Excellence for Drug Preclinical Studies (CE-DPS), Pharmaceutical and Fermentation Industry Development Center, City of Scientific Research & Technological Applications (SRTA-city), New Borg El Arab, Alexandria, Egypt
[5] Research Projects unit, Pharos University in Alexandria; Canal El Mahmoudia Street, Beside Green Plaza Complex 21648, Alexandria, Egypt.

*Correspondence: ahmedrabee@alexu.edu.eg, mohamedhaggar@gmail.com

Serial	content	page
**1.**	Copy of NMR spectra	
	Figure S1. ^{1}HNMR of **1**	**S2**
	Figure S2. ^{13}C NMR of **1**	**S3**
	Figure S3. FT-IR of compound **1**	**S4**
	Figure S4. ^{1}HNMR of **3a**	**S5**
	Figure S5. ^{13}C NMR of **3a**	**S6**
	Figure S6. FT-IR of compound **3a**	**S7**
	Figure S7. ^{1}HNMR of **3b**	**S8**
	Figure S8. D_2O-^{1}HNMR of **3b**	**S9**
	Figure S9. ^{13}C NMR of **3b**	**S10**
	Figure S10. FT-IR of compound **3b**	**S11**
	Figure S11. ^{1}HNMR of **3c**	**S12**
	Figure S12.D_2O-^{1}HNMR of **3c**	**S13**
	Figure S13. ^{13}C NMR of **3c**	**S14**
	Figure S14. FT-IR of compound **3c**	**S15**
	3.1. Equipment and analytical techniques	**S16**

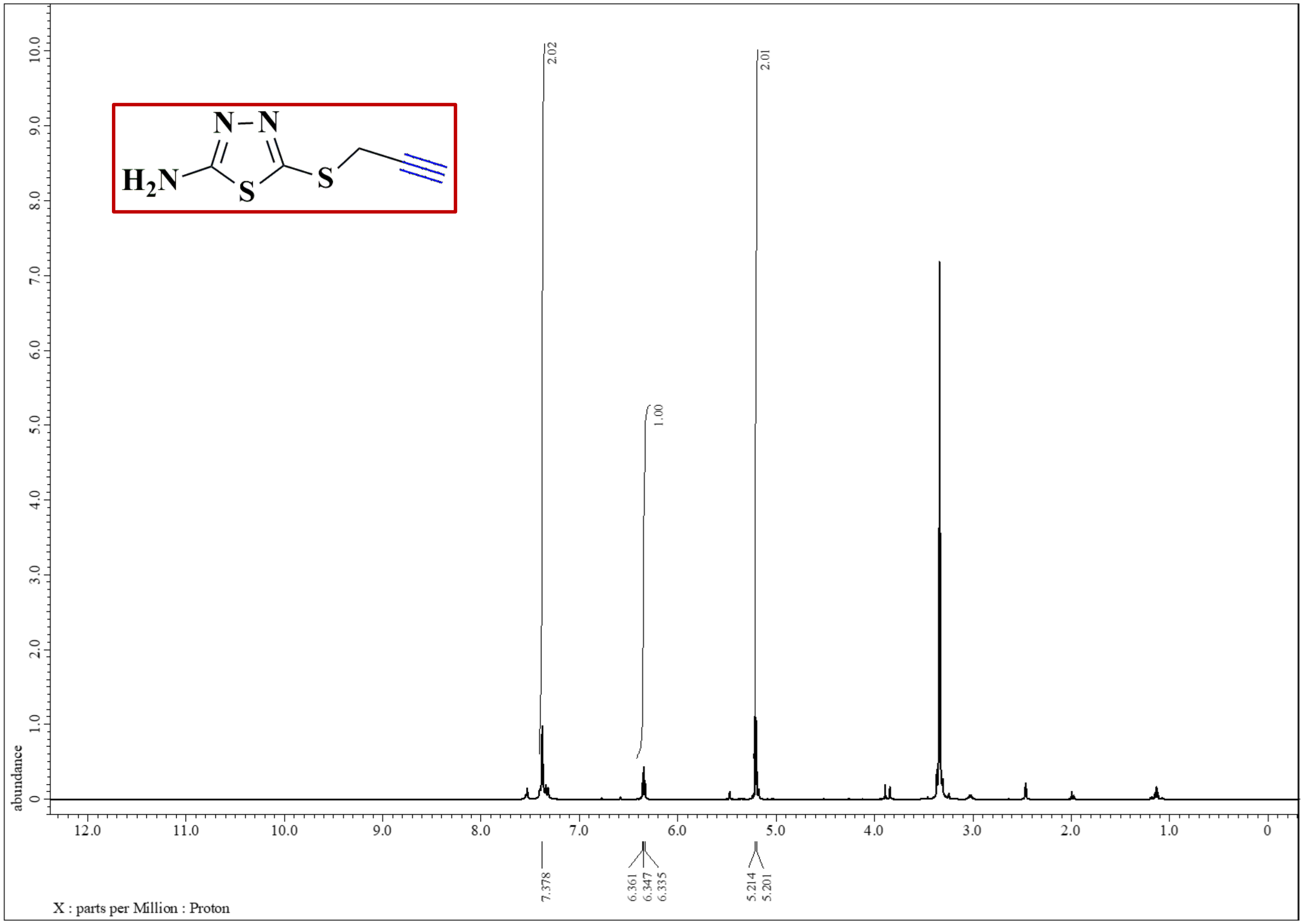

Figure S1. ^{1}H NMR of compound **1** (DMSO-d6)

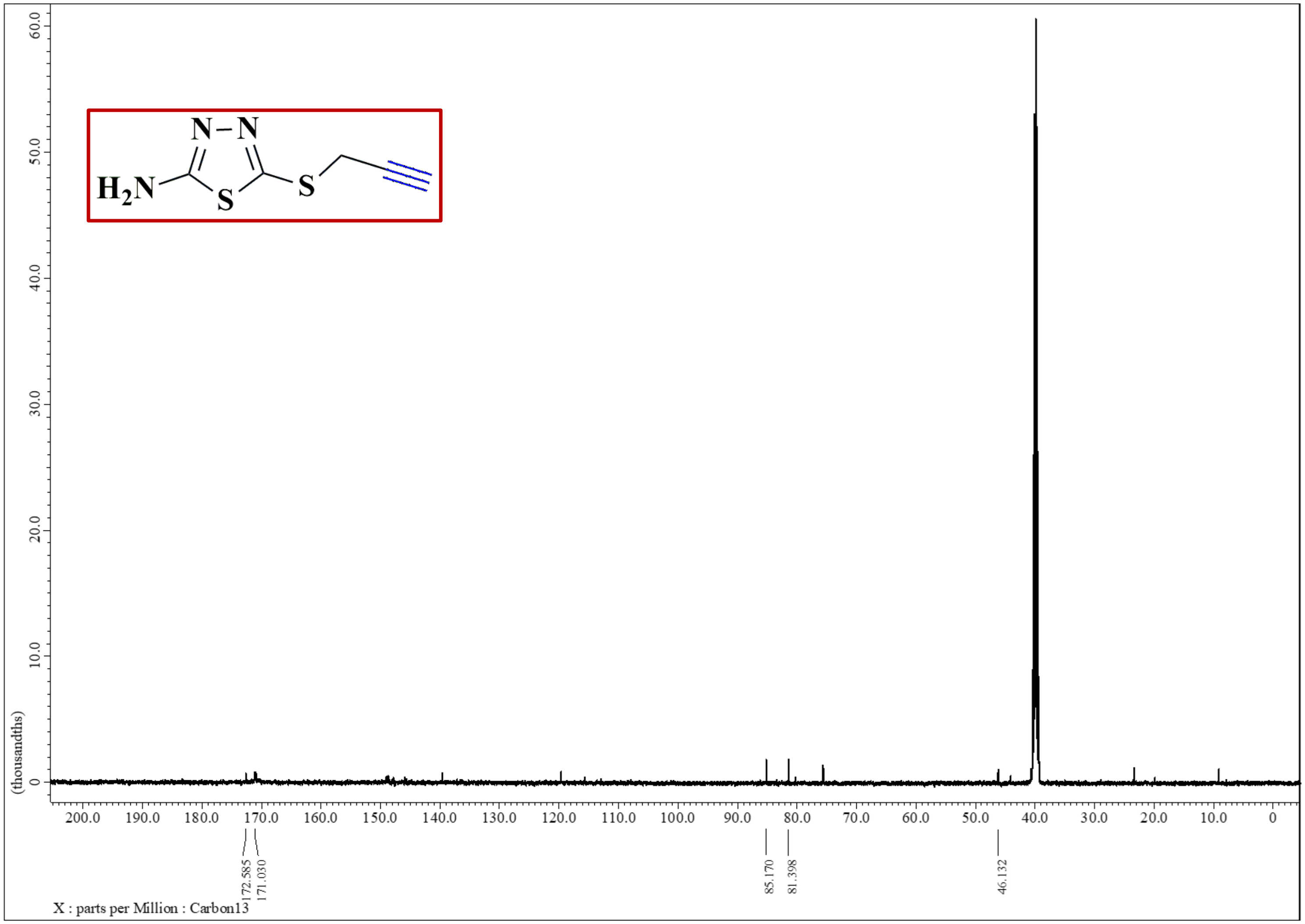

Figure S2. ^{13}C NMR of compound **3a** (DMSO-d6)

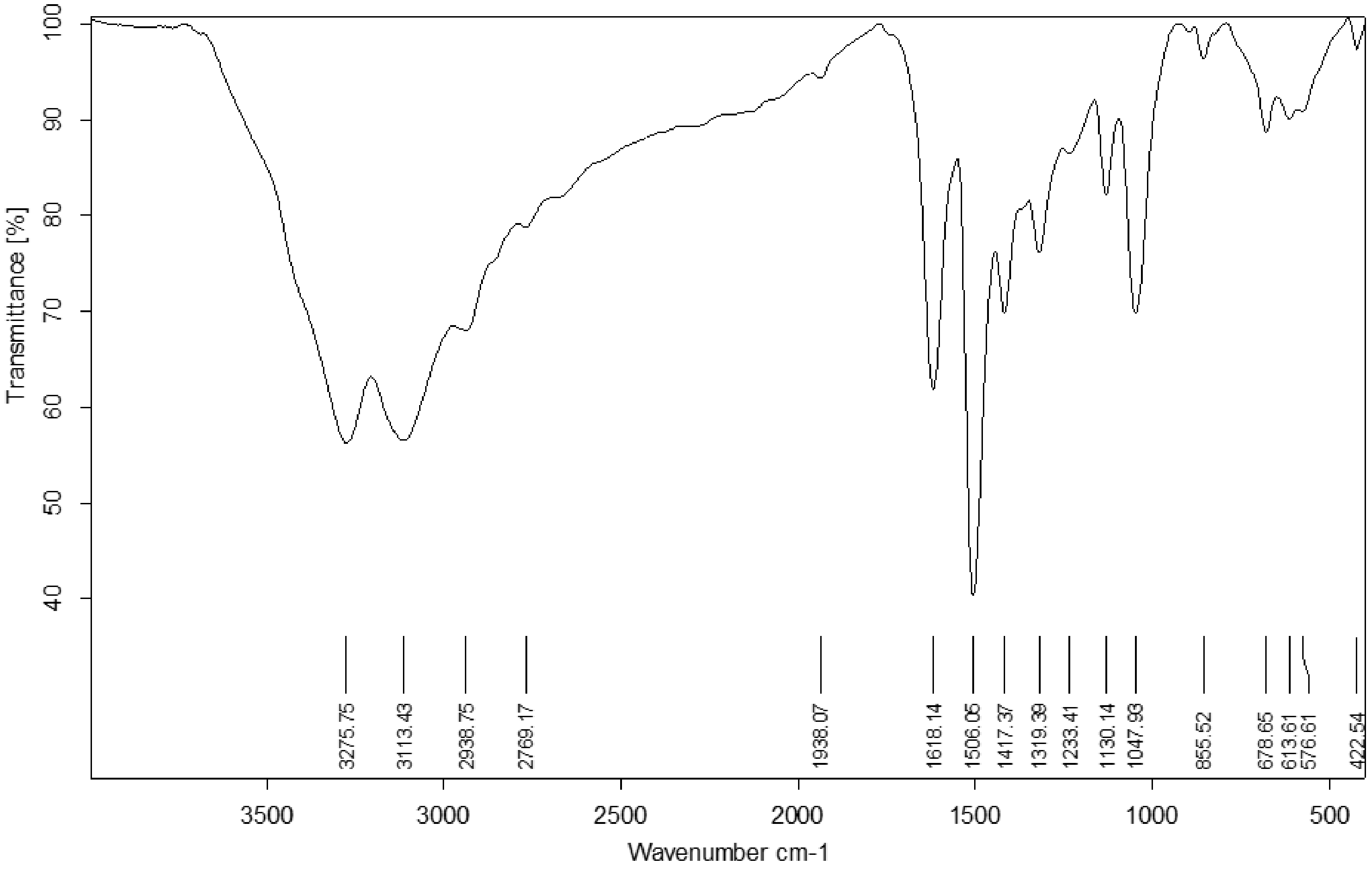

Figure S3. FT-IR spectrum of compound **1**

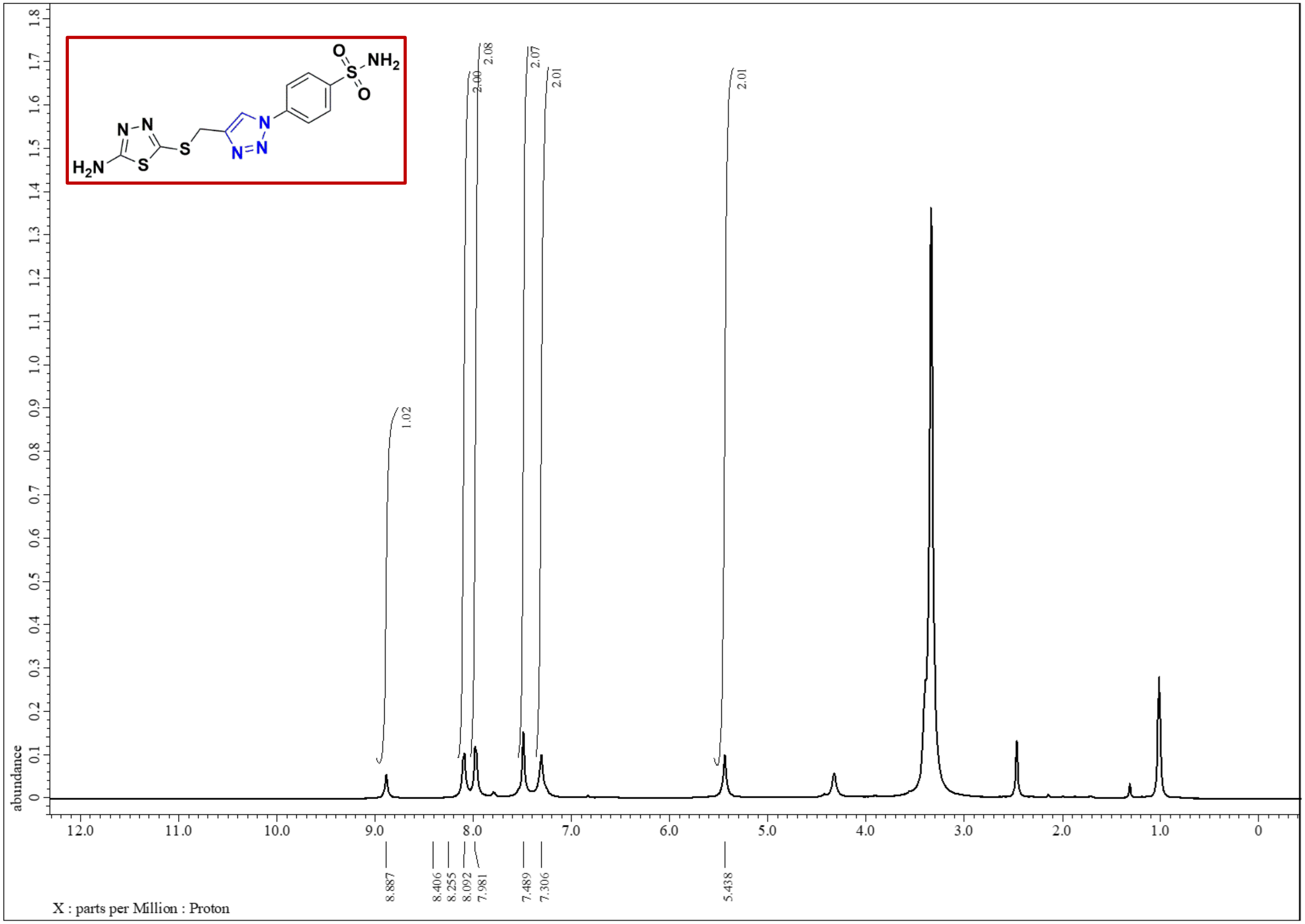

Figure S4. ^{1}H NMR of compound **3a** (DMSO-d6)

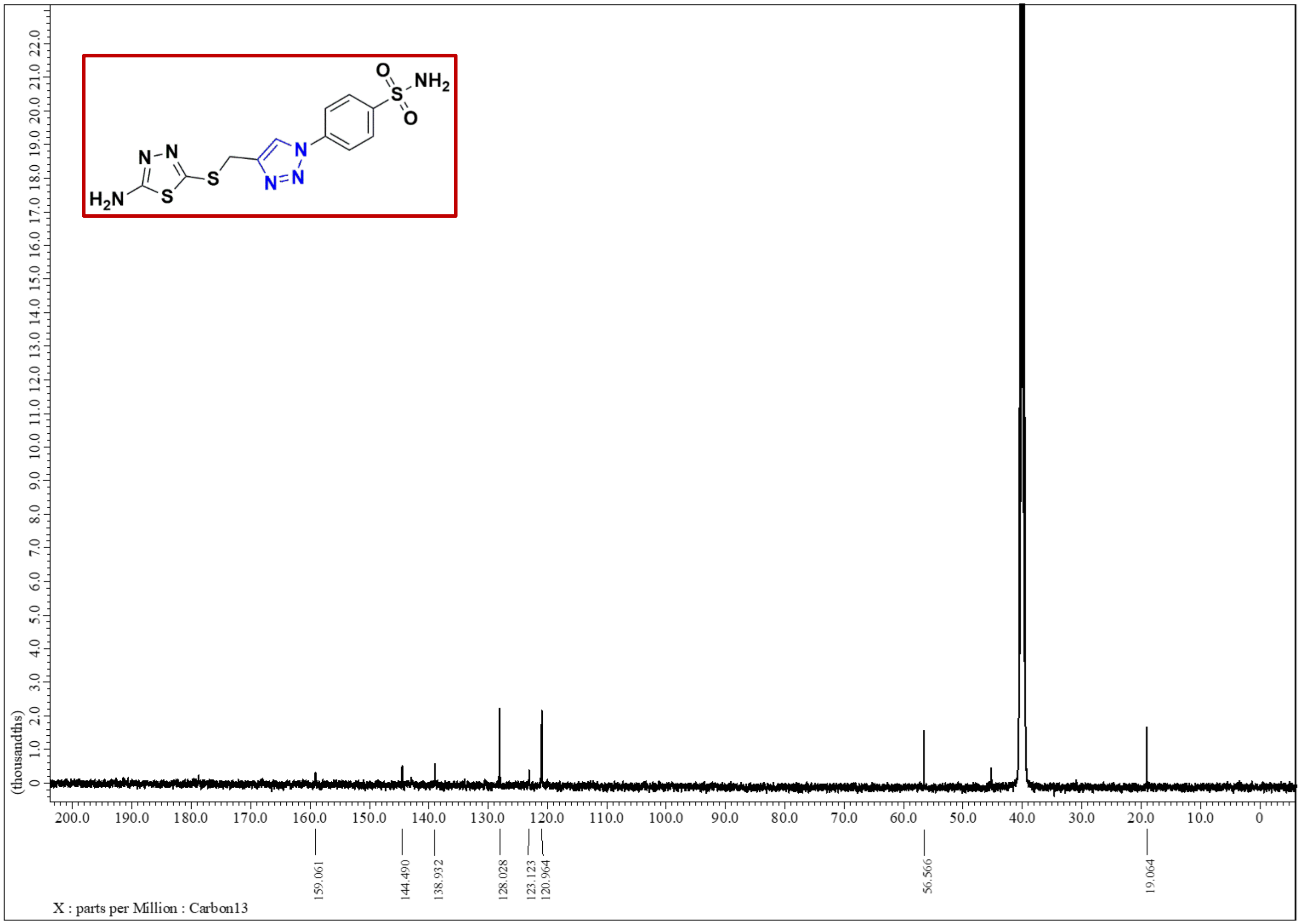

Figure S5. ^{13}C NMR of compound **3a** (DMSO-d6)

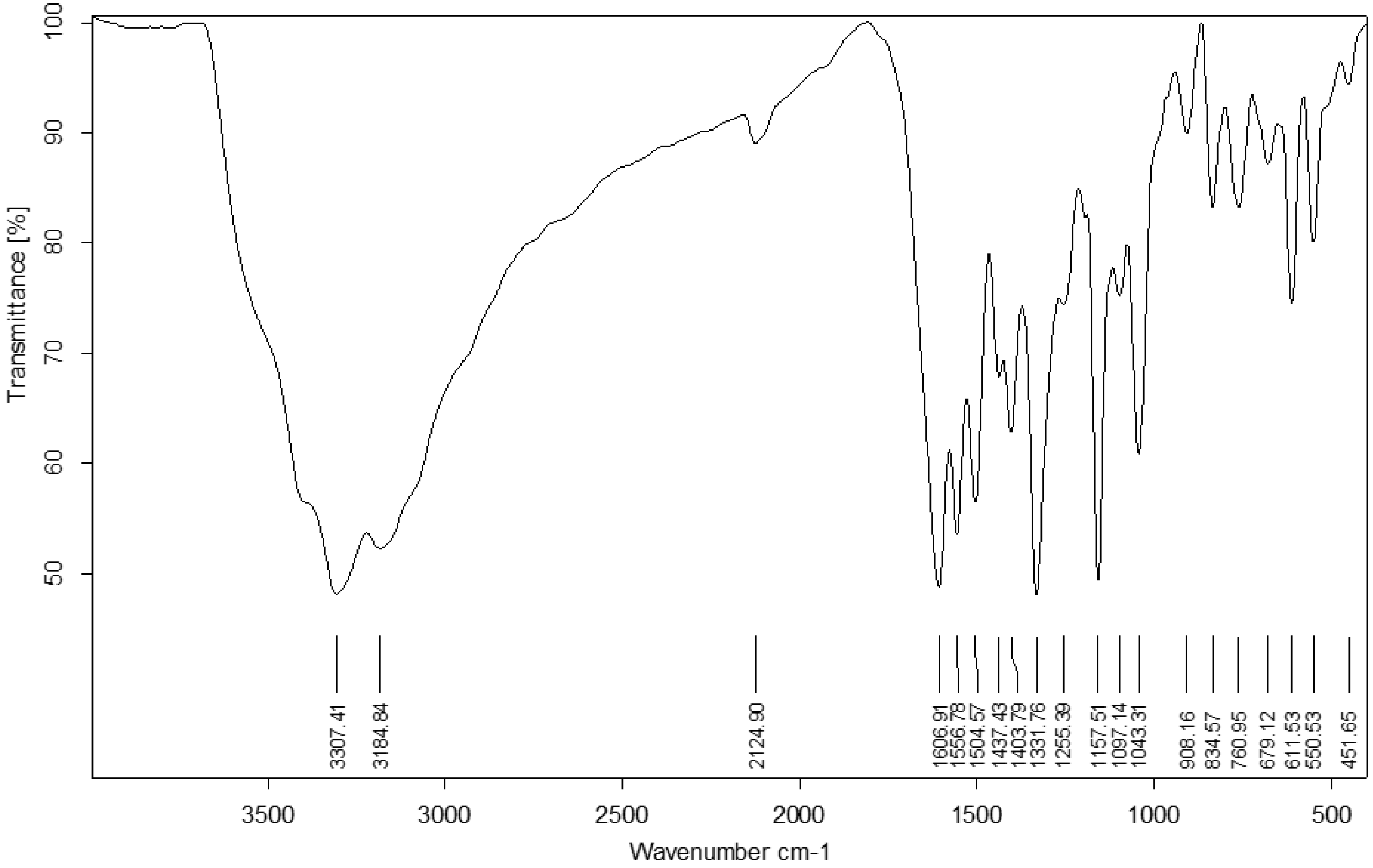

Figure S6. FT-IR spectrum of compound **3a**

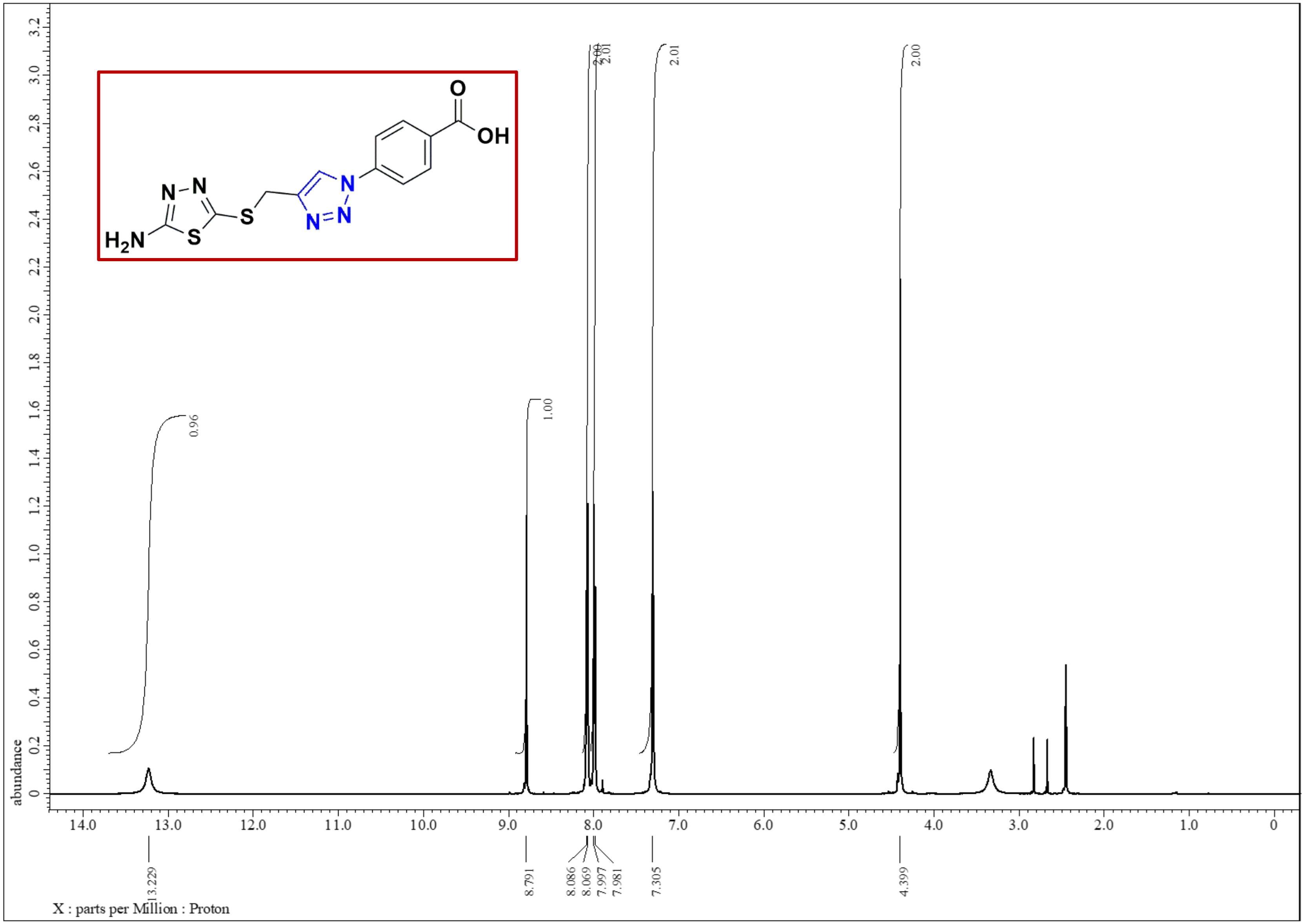

Figure S7. ^{1}H NMR of compound **3b** (DMSO-d6)

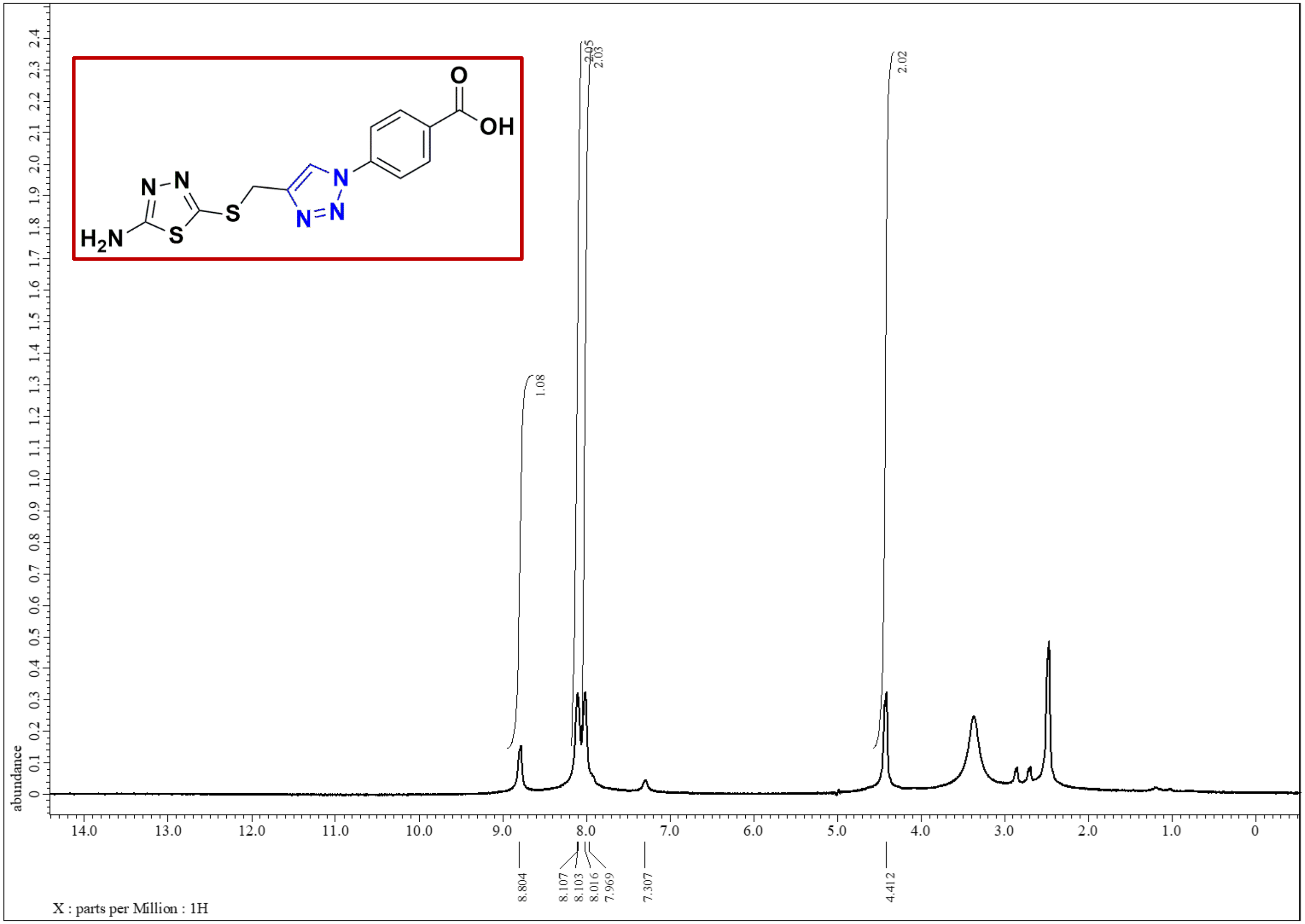

Figure S8. D_2O-^{1}H NMR of compound **3b** (DMSO-d6)

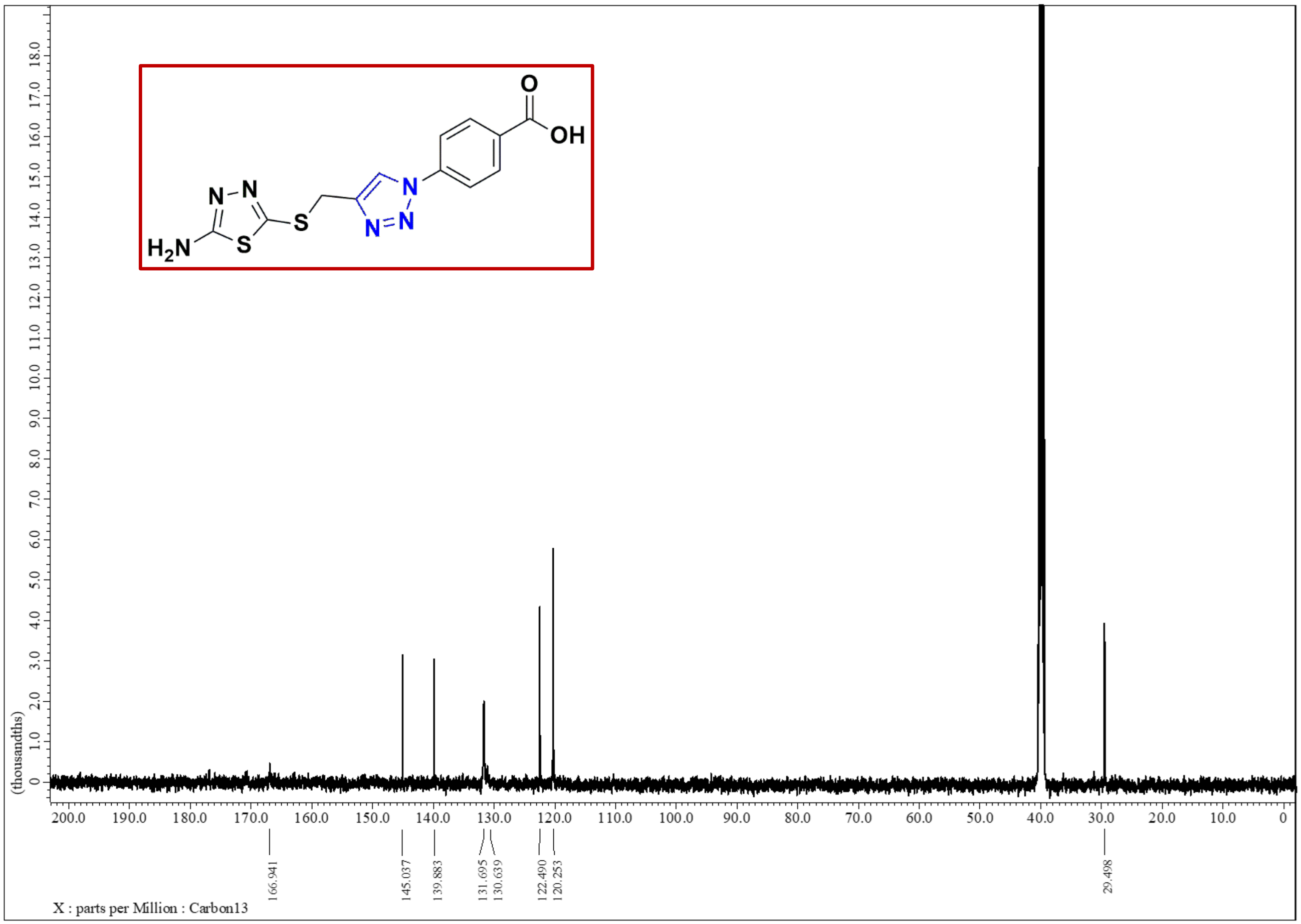

Figure S9. ^{13}C NMR of compound **3b** (DMSO-d6)

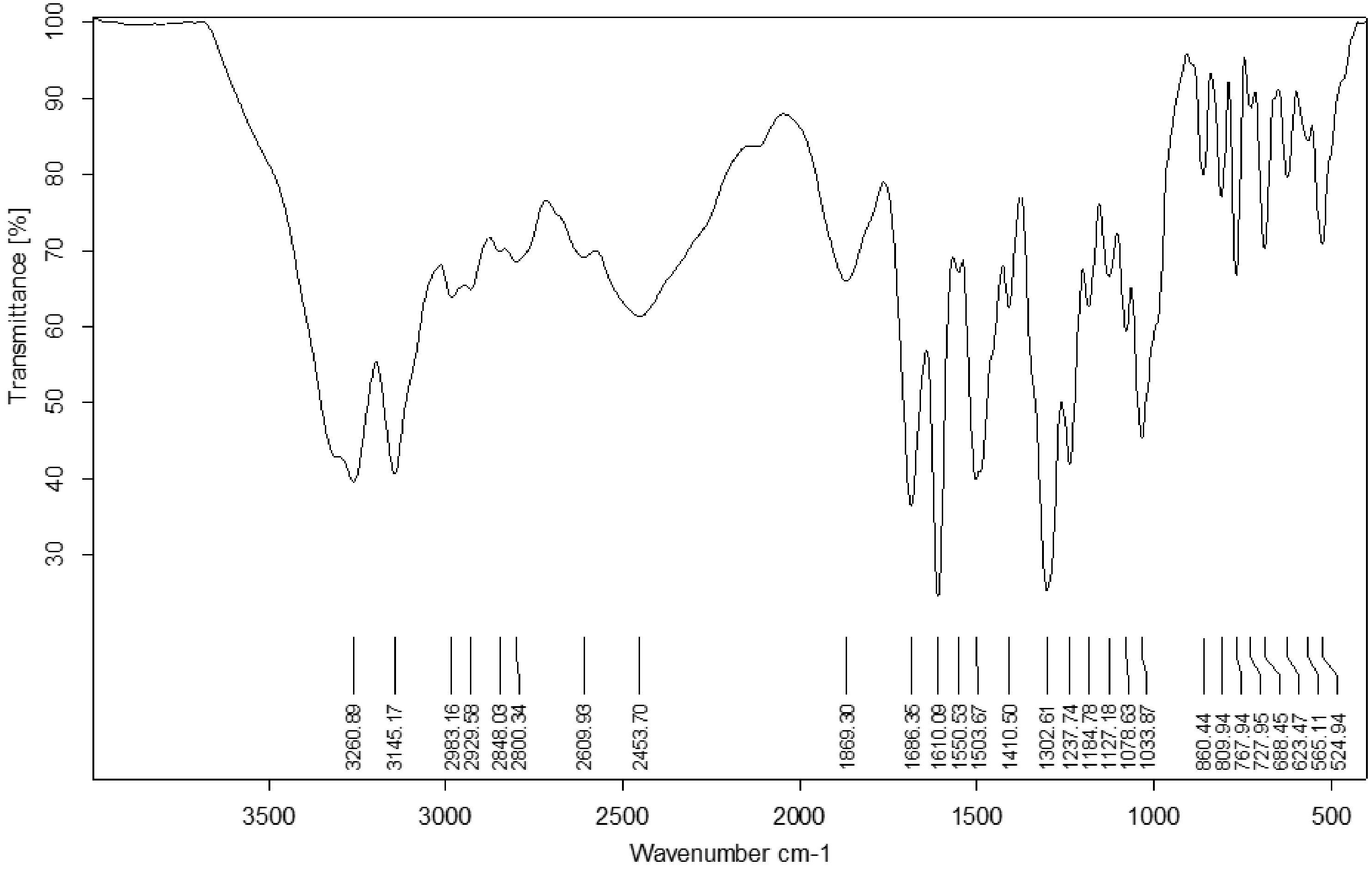

Figure S10. FT-IR spectrum of compound **3b**

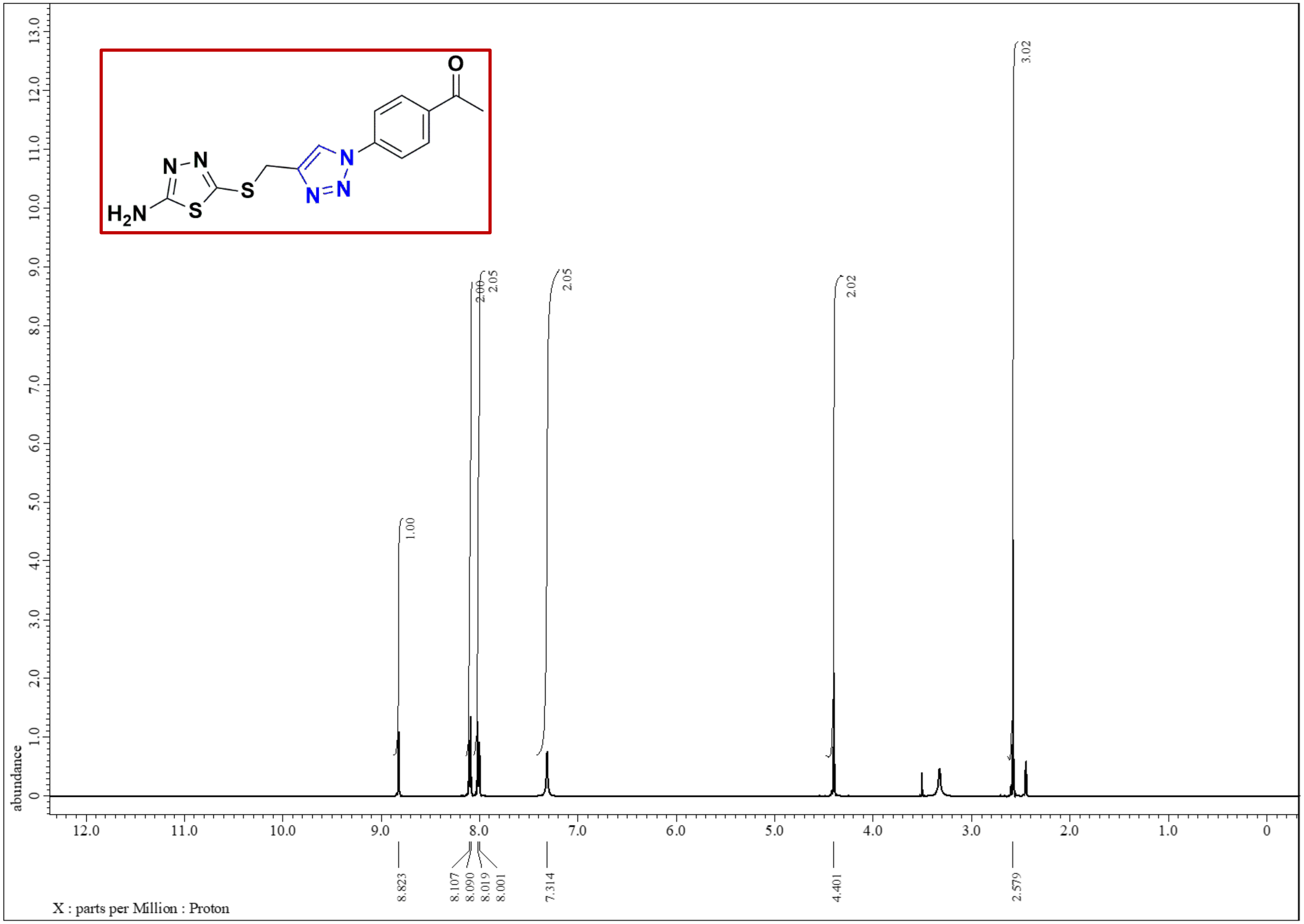

Figure S11. ^{1}H NMR of compound **3c** (DMSO-d6)

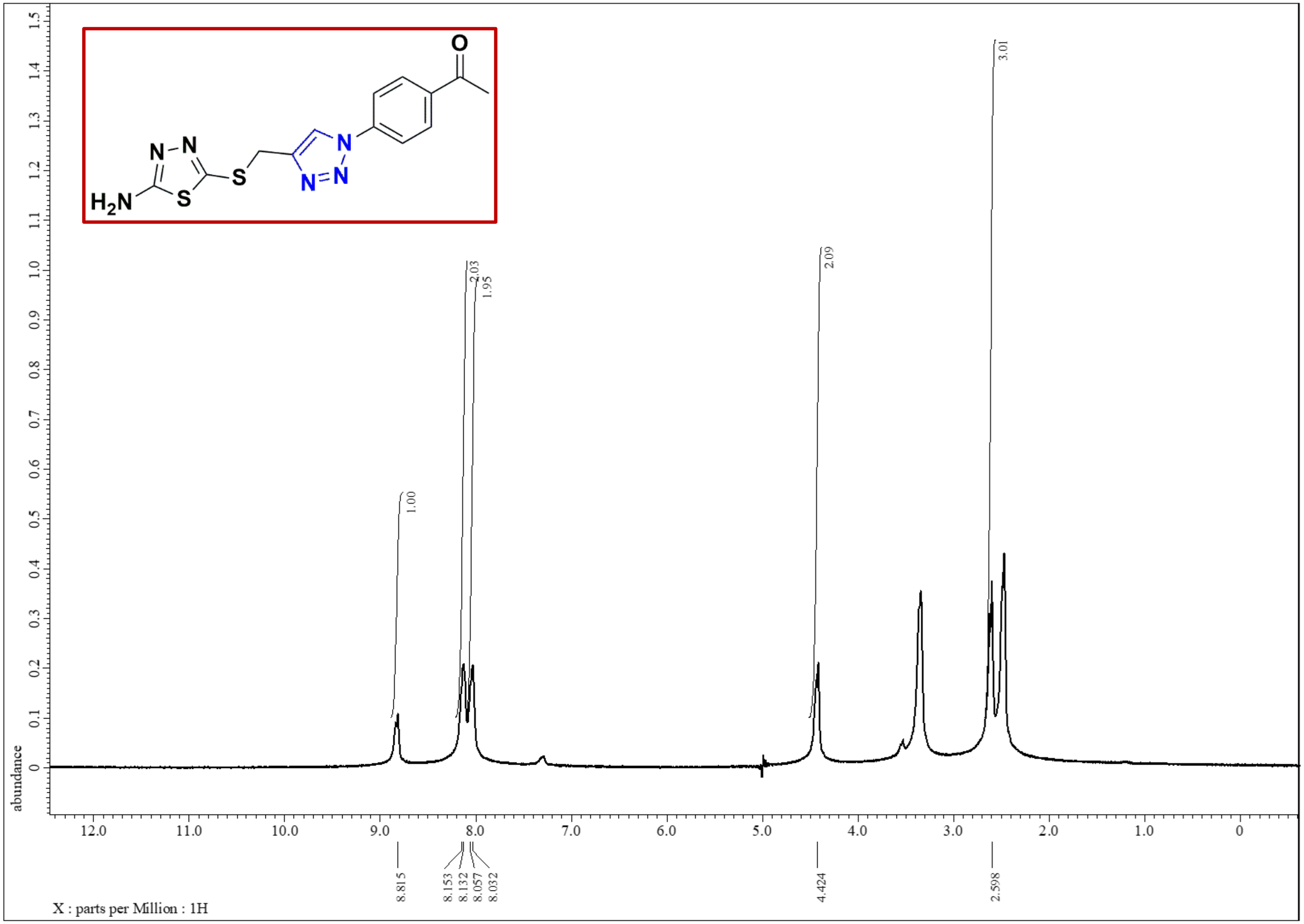

Figure S12. D_2O-^{1}H NMR of compound **3c** (DMSO-d6)

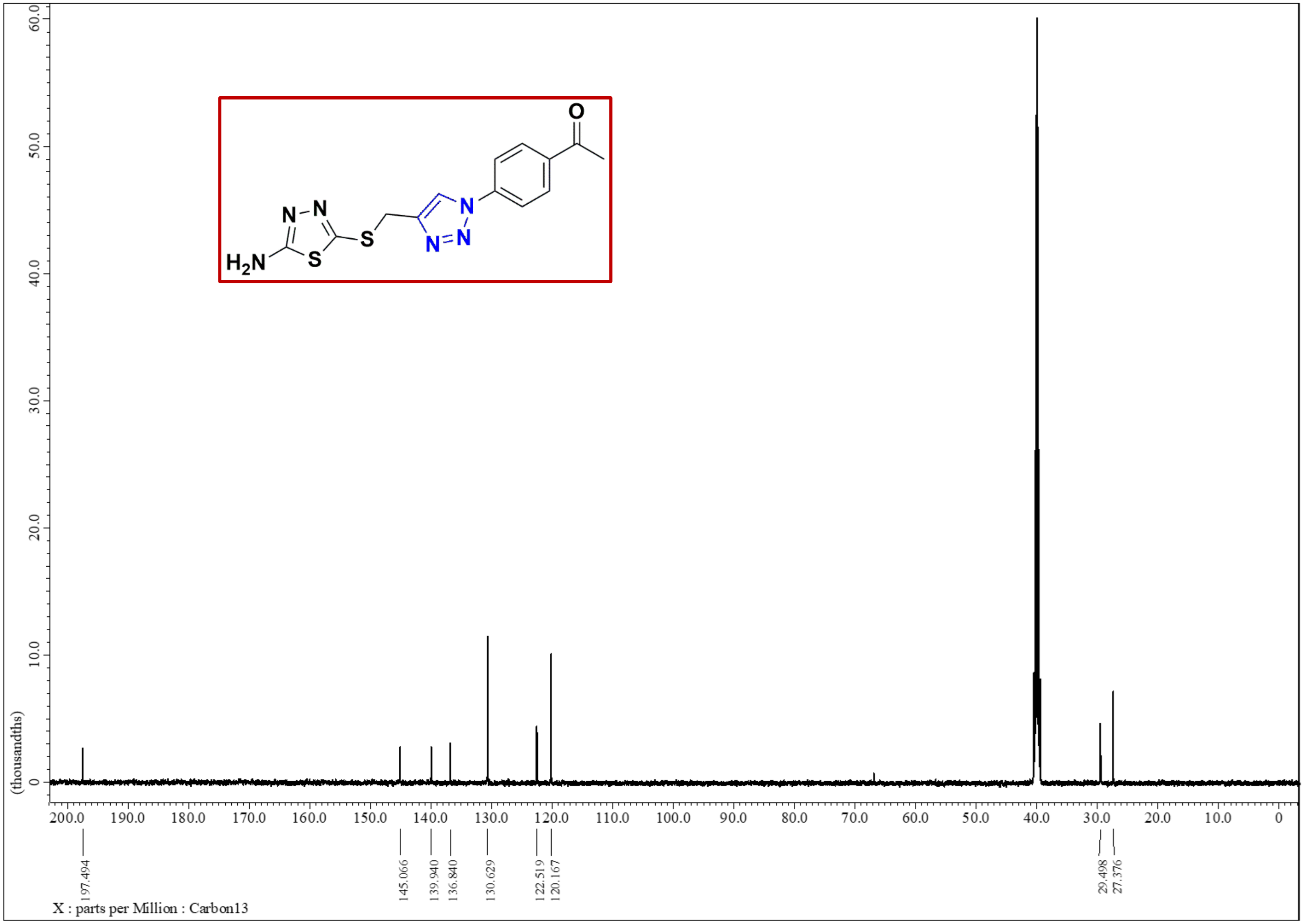

Figure S13. ^{13}C NMR of compound **3c** (DMSO-d6)

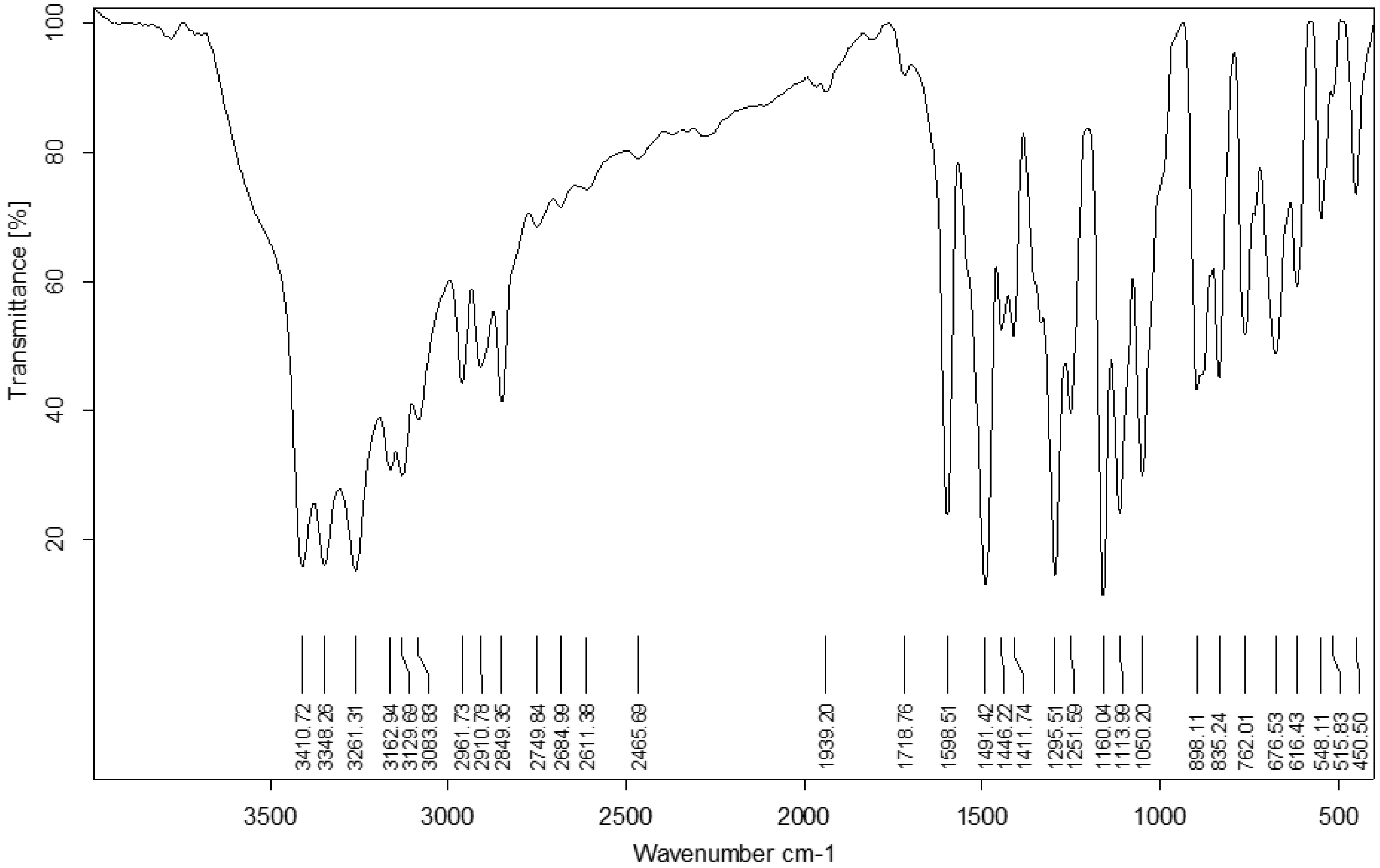

Figure S14. FT-IR spectrum of compound **3c**

Material and method

3.1. Equipment and analytical techniques

Commercially available solvents and reagents were purified according to reported standard procedures. All melting points are measured on Melt-Temp apparatus Model number 1002D, 220-240 V, 200 watts, 50/60 Hz and are uncorrected. Thin layer chromatography (TLC) was performed on aluminum plates silica gel (Fluka, 60 F254 layer thickness 0.2 mm). Visualization of the TLC during monitoring of the reaction was done by UV VILBER LOURMAT 4w-365 nm or 254 nm tube. NMR spectra measured with JEOLJNM-ECA 500 MHz spectrometer, using (DMSO-d6) solvent. Chemical shifts (δ) are given in ppm relative to the signal for TMS as internal standard, and coupling constants are quoted in Hz. 13C NMR spectra were recorded on a 100 MHz and 125 MHz spectrometer. The IR spectra were obtained using a Perkin-Elmer FT-IR spectrum BX and Bruker tensor 37 FT-IR, and wavelengths are reported in Cm^{-1}. Transmission electron microscopy (TEM) analysis was also performed at the Electron Microscope Unit of Alexandria University to evaluate the fine structural characteristics and particles size of Nano formulations.

3.2.Drug and Lead Likeness properties estimation.

SwissADME dug design free online tool (http://www.swissadme.ch/) was used for estimation of compounds physicochemical properties along with their Drug and Lead Likeness properties. To ensure compounds suitability for further consideration and screening along with ensuring absence of Pan-assay interference compounds (PAINS) alerts.

3.3. Molecular modeling and docking

Schrödinger suite Maestro 13.5.128 (LLC, New York, NY) was used to perform computational docking. Various modules were used including ligand interaction display, LigPrep, Protein Preparation Workflow, Grid Generation, Glide Docking and MM-GBSA.

3.3.1. Protein structures collection, preparation and receptor grid generation

Using Maestro's module "protein preparation workflow", PDB structures 4EY7, 7Q1O, 6N2W and 5KIR of AChE, BuChE, LOX-5 and COX-2, respectively were retrieved using "Get PDB" tool. Each structure was then prepared using the "preparation workflow" tab. All settings were set as default for preprocess step, except for enabling "filling missing loops" and "delete water beyond hits" which was adjusted at 8 A. Finally, optimization and minimization settings were kept as default including using of OPLS4 force field. Prepared protein structures and "Receptor grid generation" module was used to generate receptor grids. Receptor and its centroid were identified through selection of the co-crystalized ligand. All default settings were kept.

3.3.2. Ligands obtaining and preparation

Structures were imported to maestro, where "LigPrep" module was used for their preparation using OPLS4 force field and determining chirality from 3D structures. Ionization states were generated at pH 7.0 ± 2.0 using Epik along with desalting, generating tautomer's and at most 250 stereoisomers per ligand.

3.3.3. Molecular docking and binding free energy calculation.

Docking and estimation of binding free energy were performed using "Ligand docking" and "MM-GBSA" modules, respectively. For docking, prepared ligand structures and generated receptor grids were used along with "Extra Precision" docking protocol. For each trial, the pose showing the best docking score for each compound was used for further investigations. Docking produced complexes were used for binding free energy estimation (MM-GBSA dG bind), using VGSB solvation model and OPLS4 force field.

3.3.4. Validation of protein preparation and generated grid

For validation of both protein preparation and subsequent generated grid, the co-crystalized ligand present in the structure is exported separately through selection of the molecule followed by "export structure" found in "File" menu. Then, the structure of the same co-crystallized ligand for each protein structure was downloaded from "PubChem" database. Following retrieval, each downloaded ligand structure was prepared and docked to its respective protein structure using the same method mentioned above for ligand preparation and molecular docking. The previously exported structure was used for core pattern comparison for RMSD calculations.

3.3.5. **Frontier molecular orbitals (FMOs)**

Some parameters can be calculated from the FMOs according to the same method at the same basis sets including energy gap [(□E) = (ELUMO – EHOMO)], hardness [(η) = (ELUMO-EHOMO)/2], softness [(S) = 1/(ELUMO-EHOMO)], electronegativity [(X) = (-EHOMO-ELUMO)/2], electronic chemical potential [(μ) = -X], and fractional number of electrons transferred [(ΔN) = - μ/η].
